# Gels as adjuvant to non-surgical periodontal therapy: A systematic review and meta-analysis

**DOI:** 10.1016/j.heliyon.2023.e17789

**Published:** 2023-07-01

**Authors:** Pierre-Yves Gegout, Céline Stutz, Olivier Huck

**Affiliations:** a Université de Strasbourg, Faculté de Chirurgie Dentaire, Periodontology, Strasbourg, France; b Hôpitaux Universitaires de Strasbourg, Pôle de Médecine et Chirurgie Bucco-dentaires, Strasbourg, France; c INSERM (French National Institute of Health and Medical Research), UMR 1260, Regenerative Nanomedicine, Fédération de Médecine Translationnelle de Strasbourg (FMTS), Strasbourg, France

**Keywords:** Scaling, Root planing, Hydrogels, Local delivery, Periodontitis, Non-surgical treatment

## Abstract

**Objective:**

This systematic review and meta-analysis evaluated the effect of the use of available drugs loaded gels used as adjunct to non-surgical periodontal therapy.

**Methods:**

Systematic research on PubMed/MEDLINE, Cochrane Central register of Controlled Trials, and Embase databases up to December 2021 was performed. Randomized clinical trials (RCT) which compared the outcomes of scaling and root planing (SRP) + local adjuvant administration (gel) versus SRP + placebo or SRP alone in Humans were included. The primary outcome measures were PPD and CAL changes at 3 months.

**Results:**

After articles screening, 77 articles were included and assessed for quality. Then, a meta-analysis was conducted in studies with at least 3 months of follow-up. Clinical improvements were found to be significant for tetracyclines (−0.51 [-0.71;-0.31] *p* < 0.001), macrolides (−0.71 [-1.04;-0.38] p < 0.001), statins (−0.84 [-0.98;-0.70] *p* < 0.001), metformin (−1.47 [-1.66;-1.29] *p* < 0.001) and hyaluronan (−1.61 [-2.28;-0.94] *p* < 0.001) loaded gels, but non-significant for chlorhexidine (−0.48 [-1.10; 0.14] *p* = 0.13), metronidazole (−0.50 [-1.20; 0.20] *p* = 0.16) and bisphosphonates (−0.42 [-1.39; 0.54] *p* = 0.539) gels.

**Conclusion:**

Adjunctive use of drugs loaded gels to non-surgical periodondal treatment could improve PPD reduction at 3 months. However, huge disparities remain when comparing the outcomes of the differents drugs used. Future comparative studies should be considered to determine precisely short and long term benefits of such treatments.

## 1 Introduction

Periodontitis is a dysbiotic disease characterized by the destruction of the periodontium (alveolar bone, cementum, periodontal ligament, gingiva) and considered the main cause of tooth loss [1,2]. More than 50% of the global population is affected by periodontitis with 11% presenting a severe form of the disease [3]. Periodontitis is associated with a shift from a symbiotic periodontal microbiota to a dysbiotic one. This dysbiosis is associated with a higher prevalence of anaerobic bacteria such as *Porphyromonas gingivalis* which is considered a keystone pathogen [4] able to invade tissues and to activate the inflammatory signaling pathways to promote inflammation [5]. Periodontitis manifests itself by clinical attachment loss, increase of periodontal pocket depth (PPD), bleeding on probing (BOP) and could be associated with suppuration, pain and gingival swelling jeopardizing long-term survival of the affected teeth [6,7].

Current periodontal treatment aims to restore symbiosis and suppress inflammation of the *periodontium* and to improve clinical attachment level (CAL). Besides oral hygiene instructions and modification of the potential local and systemic risk factors, non-surgical periodontal treatment (scaling and root planing (SRP)) is the cornerstone of current treatment strategy [8,9]. However, for severe cases, the treatment outcomes might be impaired due to the difficulty of instrumental access within the lesion, the bacterial invasion of the soft tissues or the sustained inflammatory reaction emphasizing the need of adjuvant treatments including surgical approaches [10]. More recently and under certain conditions, adjuvants have been proposed to be applied even during the early steps of the periodontal therapy to promote response to periodontal treatment [11].

To reduce the indication of surgical invasive and technically demanding procedures, several adjuncts to SRP have been proposed such as the use of antibiotics or anti-inflammatory drugs. These adjunctive therapies improved treatment outcomes in terms of PPD reduction and CAL gain [12,13]. However, their systemic administration requires the use of high dose of active drugs to reach efficient concentration at the treated site, the compliance of the patient to follow the selected administration regimen and could be associated to side effects. Consequently, several local treatments such as gels, fibers or chips loaded with different active molecules or drugs have been developed and evaluated in clinical settings. The main advantages of such treatments are the delivery of active drugs at the precise site of the lesions, the reduced risk of side effects and the possibility of 3D stabilization of the blood clot [14].

Different scaffolds such as fibers, membranes or gels are used for the local delivery of molecules or drugs in the periodontal defects [[15], [16], [17], [18]]. Amongst these differents types of scaffold, gels seem to be the most convenient mode of delivery since the application within periodontal lesions does not require any advanced materials or techniques. Gels formulation and composition can also be adapted to influence gels’injectability and 3D stability through their rheological properties to influence molecules or drugs delivery [19].

The aim of this systematic review was to evaluate the efficacy of locally administered gels as adjuvant to SRP in terms of PPD reduction and CAL gain in Humans.

## 2 Methods

### 2.1 Screening and selections of papers

This systematic review complied with the PRISMA statement [20]. A literature search was performed independently by two blinded researchers (P.-Y.G., O.H.) and confirmed by another (C.S.). Relevant studies were identified from: PubMed/MEDLINE, Cochrane Central register of Controlled Trials, and Embase databases, encompassed up to December 2021. Hand searching comprised of checking bibliographic references of included articles and related review articles. The following strategy using Boolean was employed to identify papers using MesH, keywords and other free terms: ((((gel) OR (hydrogel)) AND (periodontal treatment) OR (scaling and root planing)) AND (clinical trial)) for all databases. Only articles published in English have been considered. This study aimed to answer the following PICO (Participant, Intervention, Control, Outcomes) question: “Is the adjunctive use of gels to SRP improving periodontal clinical parameters?”. (P) Participant: patients suffering from chronic form of periodontitis. (I) Intervention: adjunctive use of gels to non-surgical periodontal treatement. (C) Control: non-surgical periodontal treatment alone or with placebo gel. (O) Outcomes: evaluation of probing pocket depth and clinical attachment level changes after at least 3 months.

### 2.2 Eligibility criteria

A study was considered eligible for inclusion in this systematic review if it met the following criteria [1]: randomized controlled clinical trial (RCT) [2], assessed treatment of patients with periodontitis [3], compared SRP + local adjuvant administration (gel) versus SRP + placebo or SRP alone [4], reported results in terms of PPD or CAL changes after at least 3 months. Studies were excluded if they included patients with systemic disease, patients treated during maintenance phase and if they were duplicated or ancillary studies.

### 2.3 Outcomes

Primary outcome measures of interest were PPD and CAL changes. Secondary outcomes of interest were BOP, plaque index (PI), gingival index (GI), gingival bleeding index (GBI). Predefined data collection spreadsheets were employed for assessment of each publication. Evaluations were carried out independently and discrepancies were resolved after discussion.

### 2.4 Meta-analysis

#### 2.4.1 Risk of bias in individual studies

Risk of bias was evaluated independently by each reviewer through a process of quality analysis according to the Cochrane Reviewers’ Handbook [21]. Eventual disagreements were resolved after discussion.

#### 2.4.2 Data synthesis

The meta-analysis estimated PPD reduction expressed as the mean difference between baseline and 3 months after treatment. Heterogeneity between the studies was tested and evaluated through Q and I^2^ test. A *p*-value of Q statistic <0.1 was defined as an indicator of heterogeneity and data were considered heterogeneous for I^2^ value higher than 40%. Differences between SRP + gel and SRP+/-placebo groups were expressed as weighted mean differences (WMD) and 95% confidence interval (CI) for continuous outcomes using random models. Mean differences and standard errors were entered for each study. When data were not expressed in terms of mean differences, the mean difference was calculated as well as an estimation of the standard deviation according the Cochrane handbook instructions [22]. Indeed, standard deviation (SD) and correlation coefficient (Corr) were calculated according to these formula: ${SD}_{\begin{array}{c} E,change \end{array}}=\sqrt{{SD^{2}}_{E,baseline}+{SD^{2}}_{E,final}-\left(2\times corr\times {SD}_{E,baseline}+{SD}_{E,final}\right)}$ and ${Corr}_{e}=\frac{{SD^{2}}_{E,baseline}+{SD^{2}}_{E,final}-{SD^{2}}_{E,change}}{2\times {SD}_{E,baseline}+{SD}_{E,final}}$. When baseline, final and change SD were all unavailable, SD from another study was used as proposed by the Cochrane handbook instructions [22]. The analyzes were performed using Review Manager ((RevMan) [Computer program]. Version 5.4. The Cochrane Collaboration, 2020). Cochrane grade assessment was performed to rate the certainty of evidence of this meta-analysis and every analysis were rated either low, moderate or high (Supplemental Table 1). Cochrane sensivity analysis was also performed.

## 3 Results

### 3.1 Study selection

**Fig. 1:**
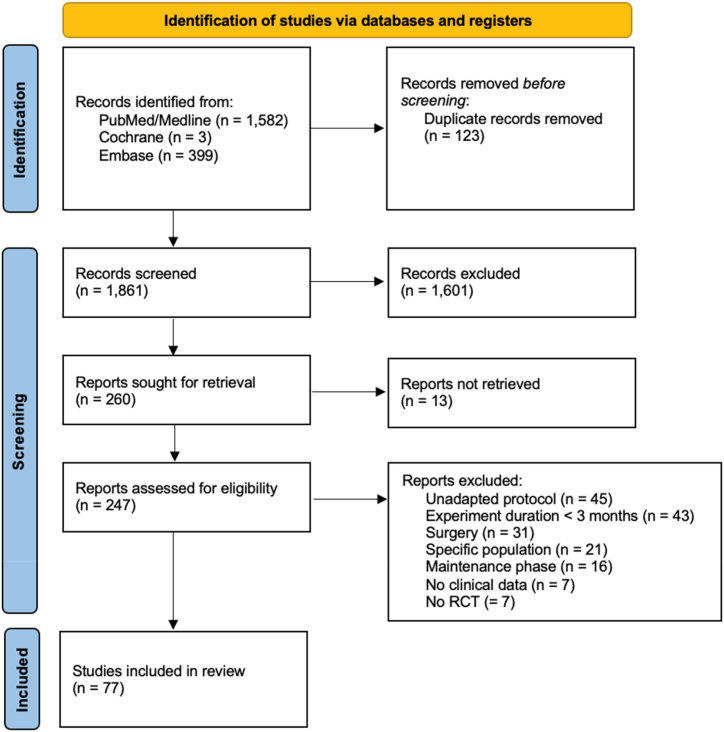
Flow diagram of literature search and inclusion.

The search strategy identified 1861 potentially relevant publications. After screening of titles and abstracts, inappropriate papers were excluded resulting in 260 publications (Fig. 1). 183 articles were excluded after full reading yielding 77 articles included in this review according to the inclusion/exclusion criteria. 40 articles were considered for meta-analysis. Screening of reviews did not give any additional information.

### 3.2 Studies characteristics

Several types of adjunctive gels have been evaluated in human RCT to treat periodontitis (Supplemental Tables 2–21). Amongst them, antiseptics such as chlorhexidine, antibiotics such as metronidazole, satranidazole, tetracyclines, macrolides or fluoroquinolones, statins, metformine, bone remodelling inhibitor such as bisphosphonates and several natural compounds including tea tree oil or green tea were evaluated. The studies were conducted according to different designs including split mouth design, quadrant based design or randomly selected sites. In the majority of the identified studies, sites with PPD>4 mm were selected. The protocols of gel application varied also significantly as, in some studies, gel was injected within the periodontal pockets only one time directly after SRP while in some others, gel application was performed immediately after SRP and at repeated sessions (3–4 application days or weeks after SRP). The follow-up ranged from 3 to 12 months. In certain studies, gel loaded with the active molecule was compared to a placebo, while in some others treatment was compared to SRP only.

### 3.3 Clinical parameters changes

#### 3.3.1 Chlorhexidine

Eleven over twelve studies evaluated the impact of treatment at 3 months [[23], [24], [25], [26], [27], [28], [29], [30], [31], [32], [33]], three at 6 months [26,31,34] and one at 9 months [23]. Four studies failed to demonstrate significant improvement associated with adjunctive use of chlorhexidine gel to SRP [23,24,32,33] while significant improvement in terms of PPD was found at 3 [25,26,28,30,31] and 6 months [26,31,34]. Moreover, CAL was found to be significantly improved in six studies at 3 [[24], [25], [26],^28^,^30^,^31^] and 6 months [26,31,34]. Significant BOP improvement [29,31] was observed at 3 months. Moreover GI were found to be significantly improved at 3 [24,30] and 6 months [34], as well as PI at 3 [30] and 6 months [34], while one study did not compared the different groups between them [27].

**Fig. 2:**
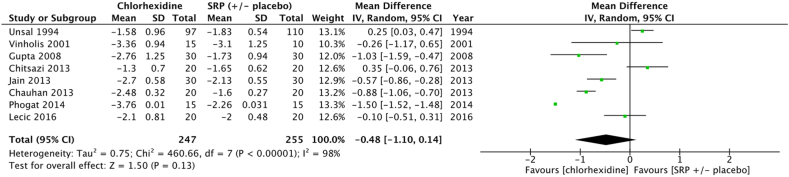
Forest plot of mean PPD reduction at 3 months at sites treated by SRP + chlorhexidine gel vs SRP ( ± placebo).

Meta-analysis failed to show a significant improvement in terms of mean PPD reduction at 3 months (−0.48 [−1.10, 0.14] *p* = 0.13) for sites treated with SRP + chlorhexidine gel vs SRP+/-placebo gel (Fig. 2) (Supplemental Tables 2 and 3).

#### 3.3.2 Antibiotics

##### 3.3.2.1 Metronidazole

Four study evaluated the effects of metronidazole loaded gel at 3 months [[35], [36], [37], [38]] while four others evaluated it at 6 months [36,37,39,40] and two at 9 months [38,41]. Significant reduction in terms of PPD was found at 6 weeks [40], 3 months [41], 6 months [37,41] and 9 months [38,41] and CAL gain at 3 [35,41], 6 [37,41] and 9 months [41] were observed. Improvement in terms of BOP at 3 [36] and 9 months was also found [38]. However, one study did not find any changes between test and control group at 6 months [39]. Different metronidazole concentrations were used amongst included studies. Most of them used 25% metronidazole gel [[35], [36], [37], [38], [39],^41^] while another one experimented 15% [40] metronidazole gel.

**Fig. 3:**
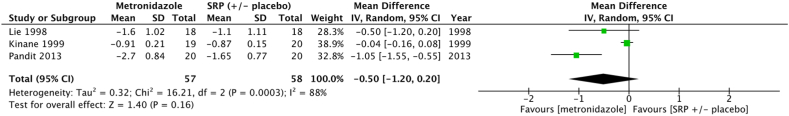
Forest plot of mean PPD reduction at 3 months at sites treated with SRP + metronidazole gel vs SRP ( ± placebo).

Meta-analysis failed to demonstrate improvement in terms of mean PPD reduction at 3 months (−0.50 [−1.20, 0.20] *p* = 0.16) for sites treated with SRP + metronidazole gel compared to SRP ( ± placebo gel) (Fig. 3) (Supplemental Tables 4 and 5).

##### 3.3.2.2 Satranidazole

Two studies evaluated the effect of satranidazole (3%) loaded gel at 6 months. These two studies presented significant PPD reduction and CAL gain at 6 months [42,43] (Supplemental Tables 6 and 7).

##### 3.3.2.3 Tetracyclines

Twelve studies evaluated the impact of tetracyclines gel at 3 months [25,[35], [36], [37],^44^,^45^,[45], [45], [46], [47], [48], [49], [50], [51]], two at 6 months [36,37,44,45,48,50] and two at 9 months [48,50]. Different molecules were used amongst these studies. Three used tetracycline alone [36,46,49] while in two others, the effect of tetracycline supplemented with citric acid was evaluated [46,49]. Three studies used minocycline 2% [37,47,48], and minocycline microspheres [35] while the remaining four evaluated doxycycline 1% [51], 10% [25,44], 15% [45], 1% doxycycline-loaded chitosan nanoparticles [51] and another one compared two test groups with doxycyline gel 10% and doxycycline microspheres [50]. An increased PPD reduction was observed at 3 months [25,35,[44], [45], [46],^48^,^49^,^51^], 6 [44,45] and 9 months [48]. Significant CAL gain was also observed at 3 [25,35,44,46,47,49,51] and 6 months [44]. Other parameters such as PI [49], GI [45,49,51] at 3 months, or BOP at 3 months [47] and 6 months [44] were found to be improved. However, Lie et al. [36] did not find any significant differences between SRP + tetracycline gel and SRP ( ± placebo), while two other studies did not present any statistical analysis [37] or did not perform intergroups comparison [50].

Meta-analysis showed significant improvement in terms of mean PPD reduction at 3 months for sites treated with SRP + tetracyclines (−0.51 [−0.71, −0.31] *p* < 0.001). Number of sites evaluted in the included studies ranged from 10 to 260 sites.

In minocycline subgroup, meta-analysis showed significant improvement in terms of mean PPD reduction at 3 months (−0.64 [−0.97, −0.30] *p* < 0.001) for sites treated with SRP + minocycline gel compared to SRP ( ± placebo gel). For tetracycline subgroup, meta-analysis showed significant improvement of mean PPD reduction at 3 months (−0.44 [−0.74, −0.13] *p* < 0.001) for sites treated with SRP + tetracycline gel compared to SRP ( ± placebo gel).

**Fig. 4:**
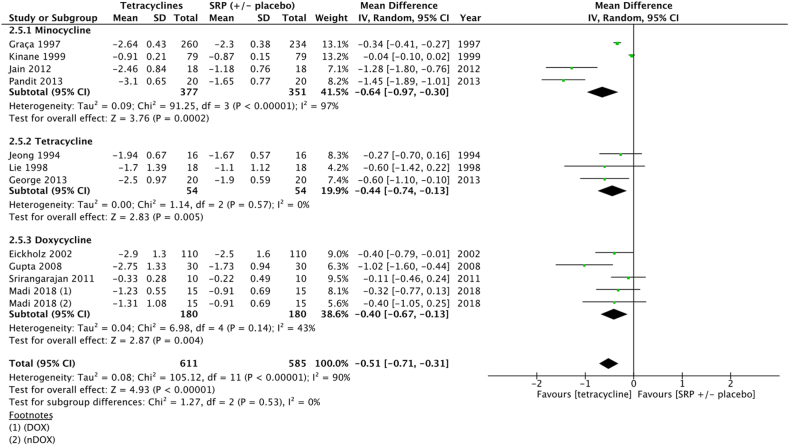
Forest plot of mean PPD reduction at 3 months at sites treated with SRP + tetracyclines vs SRP ( ± placebo).

In doxycycline subgroup, meta-analysis demonstrated significant improvement (−0.40 [−0.67, - 0.13] *p* = 0.004) for sites treated with SRP + doxycycline gel compared to SRP ( ± placebo gel) (Fig. 4) (Supplemental Tables 8 and 9).

##### 3.3.2.4 Macrolides

Regarding the effects of macrolide loaded gel on periodontal healing, studies evaluated the results at 3 months [[52], [53], [54], [55]], three at 6 months [[53], [54], [55]] and one at 9 months [54]. All studies focusing on the effect of macrolide containing gels on periodontal healing showed significant improvement in terms of PPD reduction at 3 [[52], [53], [54], [55]], 6 [[53], [54], [55]] and 9 months [54]. They also observed significant CAL improvement at 3 [[52], [53], [54]], 6 [[53], [54], [55]] and 9 months [54]. Interestingly, some authors found significant improvements of PI [53,54] and GI at 6 months [53] and PI at 9 months [54]. Different drugs were used amongst these studies. Two used azithromycin [52,54], one clarithromycin [53] and another clindamycin [55].

Meta-analysis showed significant improvement in terms of mean PPD reduction at 3 months (−0.71 [−1.04, −0.38] *p* < 0.001). Number of sites evaluted in these different studies ranged from 25 to 40 sites.

**Fig. 5:**
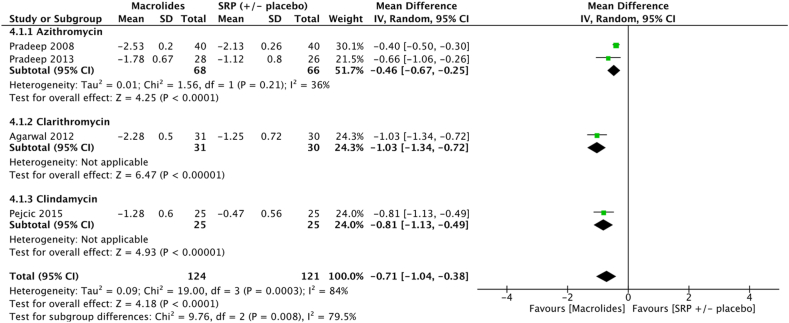
Forest plot of mean PPD reduction at 3 months at sites treated with SRP + macrolides vs SRP ( ± placebo).

In azithromycin subgroup, meta-analysis showed significant improvement in terms of mean PPD reduction at 3 months (−0.46 [−0.67, −0.25] *p* < 0.001) for sites treated with SRP + azithromycin’s gel compared to SRP ( ± placebo gel) (Fig. 5) (Supplemental Tables 10 and 11).

##### 3.3.2.5 Fluoroquinolones

Two studies evaluating the effects of fluoroquinolones on periodontal healing observed significant improvements in terms of PPD at 3 months [56,57]. Moreover, improvements of PI, GI and CAL at 3 months were measured [57]. One study evaluated the effect moxifloxacine at different concentrations [56] while another evaluated the effect of a mixture of moxifloxacine and ibuprofen [57] (Supplemental Tables 12 and 13).

##### 3.3.2.6 Statins

For the studies evaluating the effect of statins on periodontal healing, nine studies evaluated the results at 3 months [[58], [59], [60], [61], [62], [63], [64], [65], [66]], eleven at 6 months [[58], [59], [60], [61], [62],^64^,^65^,[67], [68], [69], [70]], five at 9 months [59,60,62,64,69] and one at 12 months [70].

A majority of studies found significant improvement for test group compared to control group for PPD [59,61,62,[64], [65], [66], [67],^69^,^70^] and CAL [59,61,62,64,65,67,69,70]. Hence, two studies did not compare the test and control groups [58,60] while another did not find any significant differences [63]. Differents drugs were used in these studies such as simvastatin [58,63,64,67], atorvastatin [59,60,62,65] and rosuvastatin [61,[68], [69], [70]].

Meta-analysis showed significant improvements in terms of mean PPD at 3 months (−0.84 [−0.98, −0.70] *p* < 0.001) for sites treated with SRP + statin gel compared to SRP ( ± placebo gel). In atorvastatin subgroup, meta-analysis showed significant improvement in terms of mean PPD reduction at 3 months (−0.75 [−0.87, −0.63] *p* < 0.001) for sites treated with SRP + atorvastatin gel compared to SRP ( ± placebo gel). For simvastatin subgroup, meta-analysis showed significant improvement in terms of mean PPD reduction at 3 months (−0.92 [−1.15, −0.70] *p* < 0.001) for sites treated with SRP + simvastatin gel compared to SRP ( ± placebo gel).

##### 3.3.2.7 Metformin

All studies used metformin 1% gel and observed significant improvements in term of PPD reduction, CAL gain, and intrabony defect (IBD) fill [[70], [71], [72], [73], [74], [75]]. Some of them also observed significant improvements in terms of mSBI [64,[72], [73], [74]] or PI [74].

Meta-analysis showed significant improvement in terms of mean PPD reduction at 3 months (−1.47 [−1.66, −1.29] *p* < 0.001) for sites treated with SRP + metformin gel compared to SRP ( ± placebo gel) (Fig. 7) (Supplemental Tables 16 and 17).

##### 3.3.2.8 Bisphosphonates

In studies which evaluated the potential effect of bisphosphonates on periodontal healing, four studies evalueted the results at 3 months [60,[76], [77], [78]], six at 6 months [60,[76], [77], [78], [79], [80]] and one at 9 months [60].

Except for Dutra et al., who did not find any significant differences in terms of PPD reduction and CAL gain at 3 and 6 months [76], all studies found significant PPD and CAL improvements at 3 [60], 6 [60,77,79,80] and 9 months [60]. Only Sheokand et al. found significant PPD and CAL improvements at 3 months in non-smoker patients [78]. All studies except one who used 0.05% zoledronate [77], used 1% alendronate dose [60,76,[78], [79], [80]].

Meta-analysis failed to show significant improvements in terms of mean PPD reduction at 3 months (−0.42 [−1.39, 0.54] *p* = 0.39) for sites treated with SRP + bisphosphonate gel compared to SRP ( ± placebo gel). In alendronate subgroup, meta-analysis failed to show significant improvement in terms of mean PPD reduction at 3 months (−0.32 [−1.43, 0.79] *p* = 0.57) for sites treated with SRP + alendronate gel compared to SRP ( ± placebo gel). (Fig. 8) (Supplemental Tables 18 and 19).

##### 3.3.2.9 Others molecules

For the studies which evaluated the potential effects of other molecules or compounds contained in gels, three used hyaluronan [[81], [82], [83]], tea derived products [[84], [85], [86]], *Embilica Officinalis* [87], lemongrass (2%) essential oil [88], lycopene (2%) [89], spirulina [90], herbal gel [91], Nano-Bio Fusion gel [92], *Garcinia mangostana* gel [93], boric-acid [94], curcumin (1%) [95], coenzyme Q10 [96], erythropoietin [97], melatonin [98] and grape seed extract [99]. Different concentrations of hyaluronan gel were used. Two studies used 0.8% hyaluronan gel [82,83] and one study 0.2% hyaluronan gel [81].

All studies, except four [84,91,95,96] found significant improvements in terms of PPD reduction and CAL gain in test group, while one [85] only found significant improvement for CAL at 6 months.

Meta-analysis showed significant improvement in terms of mean PPD reduction at 3 months (−1.61 [−2.28, −0.94] *p* < 0.001) for sites treated with SRP + hyaluronan’s gel compared to SRP ( ± placebo gel) (Fig. 9) (Supplemental Tables 20 and 21).

## 4 Discussion

To improve non-surgical periodontal treatment outcomes, several local therapeutics have been developed. In this review, a focus was made on the local delivery of gels loaded with drugs. The development of such adjunctive therapy is of interest especially in the management of deep lesions (PPD>5 mm + BOP), furcation lesions or refractory sites [8,100].

Nowadays, SRP is considered the gold standard of periodontitis management as it demonstrated efficacy to reduce PI, BOP and PPD and to induce CAL gain [101]. However, it is also established that, besides systemic factors, local factors associated to the 3D configuration of the periodontal lesion influence the SRP outcomes [8,102]. Therefore, to improve, either the elimination of subgingival biofilms and to restore tissue homeostasis, several types of drugs or compounds have been tested through local or systemic delivery. Regarding the local delivery of active molecules at the periodontal lesion sites, anti-inflammatory drugs, antibiotics and antiseptics were proposed and evaluated. Indeed, anti-inflammatory and anti-infectious drugs such as tetracyline, minocycline or chlorhexidine were the most tested in animal models and in clinical trials when administered systemically and locally [103]. However, their use is not yet recommended due to heterogenous results [10]. Only the use of chlorhexidine mouthwash has been suggested as an additional help for plaque management during etiological phase of periodontal treatment [10].

The main limitations related to the use of local delivered adjunctive therapy are the stability of the gel within the defect, the quantity of active drug available at site and the duration of the drug release. Several hydrogels have been tested (chitosan, xanthan, hyaluronic acid, …), however these gels do not exhibit long-term 3D stability as compared to *in-situ* forming gel [104]. In the included studies, as gels were injected within the periodontal pocket, no control of their time of contact was performed. Therefore, in several studies, gel application was repeated at 1 week interval for several times. This could be seen as an inconvenient as it needs high availabiliy and compliance of the patient.

Moreover, in most of the studies, the duration of follow-up was comprised between 3 months and 1 year. This follow-up period is in accordance with the time needed to reach a stable healing. However, it is of importance to mention that pocket closure was not considered as a study endpoint. It would be an effective measure to allow for an objective comparison among studies and to assess more precisely clinical efficacy of the tested treatment. Most of the studies included showed high heterogeneity between them, reinforcing the fact that these results need to be further confirmed.

**Fig. 6:**
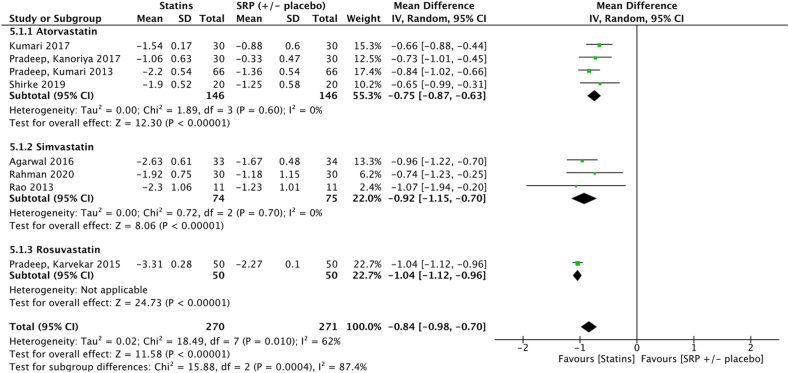
Forest plot of mean PPD reduction at 3 months at sites treated with SRP + statin gel vs SRP ( ± placebo).

**Fig. 7:**
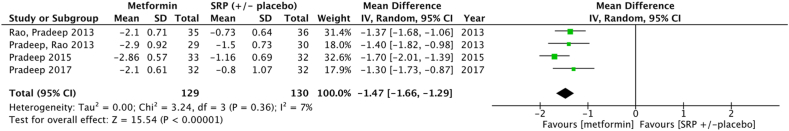
Forest plot of mean PPD reduction at 3 months for periodontal sites treated with SRP + metformin vs SRP ( ± placebo).

**Fig. 8:**
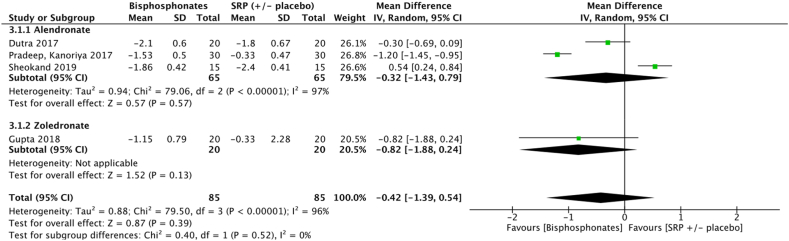
Forest plot of mean PPD reduction at 3 months for sites treated with SRP + bisphosphonates gel vs SRP ( ± placebo).

**Fig. 9:**
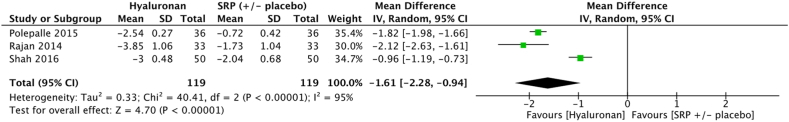
Forest plot of mean PPD reduction at 3 months at sites treated with SRP + hyaluronan gel vs SRP ( ± placebo).

Results of this meta-analysis support the clinical benefit in terms of PPD reduction for some adjuvant gels at 3 months. Amongst the eight molecules identified for meta-analysis, only chlorhexidine (−0.49 [−1.13, 0.14] *p* = 0.05), metronidazole (−0.17 [−0.46; 0.12] *p* = 0.25) and bisphosphonates (−0.32 [−1.39; 0.75] *p* = 0.56) failed to show any significant benefit at 3 months (Fig. 2, Fig. 3, Fig. 8). However, the five other molecules, i. e tetracyclines (−0.46 [−0.63;-0.29] *p* < 0.001), macrolides (−0.73 [−1.00;-0.45] p < 0.001), statins (−0.82 [−0.94;-0.69] *p* < 0.001), metformin (−1.47 [−1.71;-1.24] *p* < 0.001), hyaluronan (−1.61 [−2.31;-0.90] *p* < 0.001), demonstrated to significantly reduce PPD (Fig. 4, Fig. 5, Fig. 6, Fig. 7, Fig. 9). Sensitivity test was performed and it demonstrated that the results did not differed when low quality studies were excluded from the meta-analysis.

These results are in accordance with a previous meta-analysis evaluating the impact of adjunctive use of statins gels in periodontal non-surgical treatment, since significant PPD and CAL improvements after adjunctive use of statins gels compared to SRP alone were observed [105]. However, at contrary, a meta-analysis from Zhao in 2020 demonstrated a slight PPD improvement of the adjunctive use of chlorhexidine gel during non-surgical periodontal treatment compared to SRP alone or with placebo. These results can be explained by the fact that this meta-analysis included and compared studies having strong disparities in term of patient inclusion that could have been considered as a bias [106].

## 5 Conclusion

To our knowledge, this is the first meta-analysis focused on the adjunctive use of drugs loaded gels in the context of periodontal non-surgical treatment. As mentionned, the elaboration of new galenic formulation to improve wound stability and controlled drug release may be of importance to improve the results of the non-surgical periodontal treatment. However, many factors such as cost-effectiveness, availability or safety reasons should be considered to evaluate their rationale of use and to give clear clinical indications.

## Authors contribution

All authors listed have significantly contributed to the development and the writing of this article.

## Data availability statement

The authors confirm that the data supporting the findings of this study are available within the article, its supplementary materials, and from the corresponding author, [O.H.], upon reasonable request.

## Declaration of competing interest

The authors declare that they have no known competing financial interests or personal relationships that could have appeared to influence the work reported in this paper.
