# Supplementary material for: Gels as adjuvant to non-surgical periodontal therapy: A systematic review and meta-analysis
Source: Heliyon. 2023 Jul 1;9(7):e17789. doi: 10.1016/j.heliyon.2023.e17789 (PMC10345361; doi:10.1016/j.heliyon.2023.e17789)
Supplement: Multimedia component 1 [file mmc1.docx]

**Supplemental files**

**Supplemental figure 1 – Risk of bias**


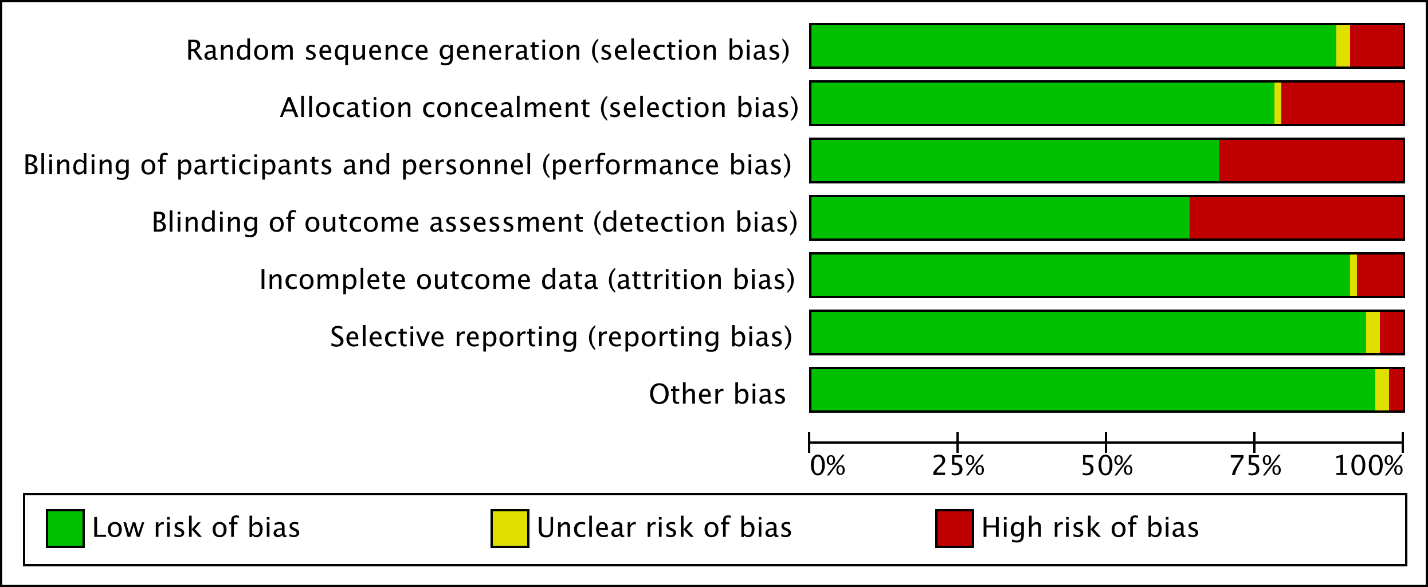


**Supplemental figure 2 – Risk of bias summary**

**
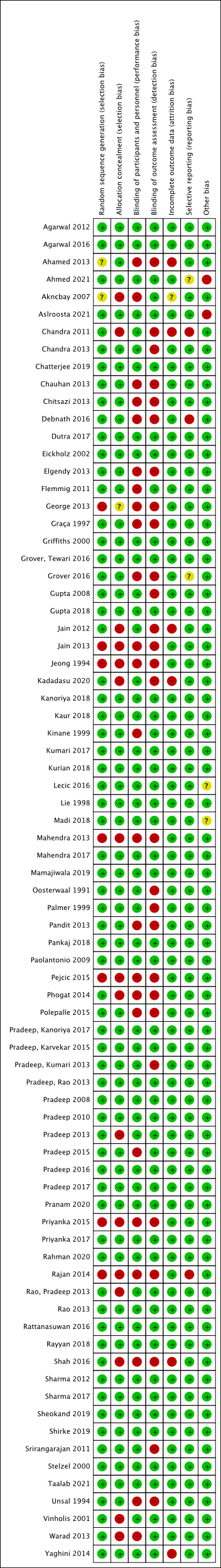
**

**Supplemental table 1 – Grade assessment**

| **Certainty assessment** | | | | | | | **№ of patients** | | **Effect** | | **Certainty** |  |
| --- | --- | --- | --- | --- | --- | --- | --- | --- | --- | --- | --- | --- |
| **№ of studies** | **Study design** | **Risk of bias** | **Inconsistency** | **Indirectness** | **Imprecision** | **Other considerations** | **Drugs** | **placebo** | **Relative (95% CI)** | **Absolute (95% CI)** |  |  |
| **Chlorhexidine (follow-up: 3 months)** | | | | | | | | | | | | |
| 8 | randomized trials | serious^a^ | serious^b^ | not serious | not serious | none | 247 | 255 | - | MD **0.48 lower** (1.1 lower to 0.14 higher) | ⨁⨁◯◯ Low |  |
| **Metronidazole (follow-up: 3 months)** | | | | | | | | | | | |  |
| 3 | randomized trials | serious^c^ | serious^b^ | not serious | not serious | none | 57 | 58 | - | MD **0.5 lower** (1.2 lower to 0.2 higher) | ⨁⨁◯◯ Low |  |
| **Tetracyclines (follow-up: 3 months)** | | | | | | | | | | | |  |
| 11 | randomized trials | serious^d^ | not serious | not serious | not serious | none | 611 | 585 | - | MD **0.51 lower** (0.71 lower to 0.31 lower) | ⨁⨁⨁◯ Moderate |  |
| **Tetracyclines - Minocycline** | | | | | | | | | | | |  |
| 4 | randomized trials | serious^a^ | not serious | not serious | not serious | none | 377 | 351 | - | MD **0.64 lower** (0.97 lower to 0.3 lower) | ⨁⨁⨁◯ Moderate |  |
| **Tetracyclines - Tetracycline** | | | | | | | | | | | |  |
| 3 | randomized trials | serious^d^ | not serious | not serious | not serious | none | 54 | 54 | - | MD **0.44 lower** (0.74 lower to 0.13 lower) | ⨁⨁⨁◯ Moderate |  |
| **Tetracyclines - Doxycycline** | | | | | | | | | | | |  |
| 4 | randomized trials | serious^e^ | not serious | not serious | not serious | none | 180 | 180 | - | MD **0.4 lower** (0.67 lower to 0.13 lower) | ⨁⨁⨁◯ Moderate |  |
| **Macrolides (follow-up: 3 months)** | | | | | | | | | | | |  |
| 4 | randomized trials | not serious | not serious | not serious | not serious | none | 124 | 121 | - | MD **0.71 lower** (1.04 lower to 0.38 lower) | ⨁⨁⨁⨁ High |  |
| **Macrolides - Azithromycin** | | | | | | | | | | | |  |
| 2 | randomized trials | not serious | not serious | not serious | not serious | none | 68 | 66 | - | MD **0.46 lower** (0.67 lower to 0.25 lower) | ⨁⨁⨁⨁ High |  |
| **Macrolides - Clarithromycin** | | | | | | | | | | | |  |
| 1 | randomized trials | not serious | not serious | not serious | not serious | none | 31 | 30 | - | MD **1.03 lower** (1.34 lower to 0.72 lower) | ⨁⨁⨁⨁ High |  |
| **Macrolides - Clindamycin** | | | | | | | | | | | |  |
| 1 | randomized trials | serious^f^ | not serious | not serious | not serious | none | 25 | 25 | - | MD **0.81 lower** (1.13 lower to 0.49 lower) | ⨁⨁⨁◯ Moderate |  |
| **Statins (follow-up: 3 months)** | | | | | | | | | | | |  |
| 8 | randomized trials | not serious | not serious | not serious | not serious | none | 270 | 271 | - | MD **0.84 lower** (0.98 lower to 0.7 lower) | ⨁⨁⨁⨁ High |  |
| **Statins - Atorvastatin** | | | | | | | | | | | |  |
| 4 | randomized trials | not serious | not serious | not serious | not serious | none | 146 | 146 | - | MD **0.75 lower** (0.87 lower to 0.63 lower) | ⨁⨁⨁⨁ High |  |
| **Statins - Simvastatin** | | | | | | | | | | | |  |
| 3 | randomized trials | not serious | not serious | not serious | not serious | none | 74 | 75 | - | MD **0.92 lower** (1.15 lower to 0.7 lower) | ⨁⨁⨁⨁ High |  |
| **Statins - Rosuvastatin** | | | | | | | | | | | |  |
| 1 | randomized trials | not serious | not serious | not serious | not serious | none | 50 | 50 | - | MD **1.04 lower** (1.12 lower to 0.96 lower) | ⨁⨁⨁⨁ High |  |
| **Metformin (follow-up: 3 months)** | | | | | | | | | | | |  |
| 4 | randomized trials | serious^f^ | serious^b^ | not serious | not serious | none | 129 | 130 | - | MD **1.47 lower** (1.66 lower to 1.29 lower) | ⨁⨁◯◯ Low |  |
| **Bisphosphonates (follow-up: 3 months)** | | | | | | | | | | | |  |
| 4 | randomized trials | not serious | not serious | not serious | not serious | none | 85 | 85 | - | MD **0.42 lower** (1.39 lower to 0.54 higher) | ⨁⨁⨁⨁ High |  |
| **Bisphosphonates - Alendronate** | | | | | | | | | | | |  |
| 3 | randomized trials | not serious | not serious | not serious | not serious | none | 65 | 65 | - | MD **0.32 lower** (1.43 lower to 0.79 higher) | ⨁⨁⨁⨁ High |  |
| **Bisphosphonates - Zoledronate** | | | | | | | | | | | |  |
| 1 | randomized trials | not serious | not serious | not serious | not serious | none | 20 | 20 | - | MD **0.82 lower** (1.88 lower to 0.24 higher) | ⨁⨁⨁⨁ High |  |
| **Hyaluronan (follow-up: 3 months)** | | | | | | | | | | | |  |
| 3 | randomized trials | serious^f^ | not serious | not serious | serious | none | 119 | 119 | - | MD **1.61 lower** (2.28 lower to 0.94 lower) | ⨁⨁⨁◯ Moderate |  |

**CI:** confidence interval; **MD:** mean difference

#### Explanations

1. Blinding of participant and outcomes bias
2. Heterogeneity
3. Blinding of participants and personnel bias
4. Randomization, blinding of participant and outcomes bias
5. Blinding outcomes bias
6. Blinding of participants, personnel, and allocation bias

**Supplemental table 2 - Characteristics of the included studies evaluating the effect of gels loaded with chlorhexidine**

| **Study** | **Design; duration** | **Sample size ; mean age; gender (M/F)** | **Clinical parameters evaluated** | **Inclusion criteria** | **Treatment** | **Local administration** | **Findings** |
| --- | --- | --- | --- | --- | --- | --- | --- |
| Oosterwaal et al., 1991 (23) | RCT  36 weeks | 10 ; 48 ; 4/6 | BOP  PPD  Presence or absence of supragingival plaque | 4 interdental pockets of 7-9 mm affecting single rooted teeth, associated with bleeding after probing, alveolar bone loss and attachment loss | SRP + placebo (methylcellulose gel 4%) Chlorhexidine gel (2%) Stannous fluoride gel (4%) Aminofluoride gel (commercially available amine-fluoride gel containing 1.25% fluoride (Elmex®, Gaba, Basel, CH)) | 3 applications of the gel within 10 minutes | No additional benefits of the gels application to mechanical debridement on clinical and microbiological outcomes. |
| Unsal et al., 1994 (33) | RCT  3 months | 22; 30-57; 10/12 | PI  GI  BI  PPD  CAL | Moderate to advanced periodontitis with 3 teeth in each quadrant with 2 sites having a PPD ≥4 mm + BOP | SRP  SRP + chlorhexidine (1%) gel  SRP + tetracycline paste | One gel and paste application after the SRP | No additional benefits of the gel application compared to SRP alone. |
| Vinholis et al., 2001 (24) | RCT  3 months | 6; 27-48; 2/4 | PI  GI  GR  BOP  PPD  CAL | Moderate to severe periodontitis.  At least 3 sites in different single-rooted teeth with 5-7 mm probing depth and BANA-positive of different quadrants. | SRP  SRP + irrigation with collagen gel  SRP + irrigation with chlorhexidine collagen-hamsana (1%) gel | Irrigation was applied after SRP and once a week during 4 weeks (0, 7, 14, 21 days) | **Improvement in clinical parameters in all groups with a significantly greater decrease in GI and bleeding in the chlorhexidine group.** |
| Gupta et al., 2008 (25) | RCT  3 months | 30; 25-75; NA | PI  GI  PPD  CAL  GM (gingival margin) | Untreated moderate to severe periodontitis or recurrent periodontitis without periodontal surgery at least for the last 24 months. A minimum of three teeth, distributed in the mouth to represent isolated experimental units (i.e., at least one tooth apart), with probing pocket depth (PPD) of 5 mm to 8 mm that bled on probing at the initial visit | SRP  SRP + doxycycline hyclate (10%) gel  SRP + xanthan based chlorhexidine (1.5%) gel | One gel application after the completion of SRP | Adjunctive use of both gels led to improved PPD compared to SRP alone. |
| Paolantonio et al., 2009 (26) | RCT  6 months | 98; 24-58; 39/59 | PI  Modified GI  BOP  Gingival recession (GR)  CAL  PPD | Moderate to advanced periodontitis with > 10 natural teeth and at least two teeth exhibiting pockets with PPD > 4 mm | SRP  SRP + xanthan-chlorhexidine digluconate/dihydroxychloride (2.5%) gel | One gel application after completion of SRP | Adjunctive use of gel improved PPD reduction and CAL gain vs SRP alone. |
| Chandra et al., 2011 (27) | RCT  3 months | 6; 20-50; NA | PI  GI  BI  RAL | Chronic generalized periodontitis. PPD measuring 5 to 7 mm in depth in different quadrants of the mouth and signs of bone loss on clinical and radiographic examination | SRP  SRP + chlosite  Chlosite | Gel application after the SRP for the group SRP + chlosite | Clinical and biological parameters improved in all the groups. |
| Chauhan et al., 2013 (28) | RCT  3 months | 60; 30-65;NA | PI  GI  PPD  CAL | Moderate to severe periodontitis.  Having at least 20 teeth, minimum of 8 teeth  with probing pocket depth (PPD) of 4‑8 mm | SRP  SRP + hyaluronan gel (H)  SRP + chlorhexidine gel (C) | Gel application after the SRP | Non significative change between test and control groups. |
| Chitsazi et al., 2013 (29) | RCT  3 months | 24; NA; 10/14 | REC  BOP  PPD  CAL | Chronic periodontitis, presence of at least one site per quadrant exhibiting pocket depth of ≥ 4 mm with BOP | SRP  SRP + xanthan-based chlorhexidine gel (CHX) | Gel application after the SRP | Only BOP was significantly different between groups at 1 and 3 months. |
| Jain et al., 2013 (31) | RCT  6 months | 30; 30-60;17/13 | PI  GI  BOP  PPD  CAL | Chronic localized or generalized periodontitis.  Presence of two periodontal sites located on the same side PD between 5 to 7 mm. | SRP  SRP + xanthan-based chlorhexidine gel | Gel application after SRP | Significant difference was found between test and control group for PPD, CAL, PI and BOP. |
| Phogat et al., 2014 (30) | RCT  3 months | 30; 30-50; NA | PI  GI  PPD  CAL | Chronic periodontitis with at least 3 nonadjacent interproximal sites with 4-8 mm of PPD | SRP  SRP + chlosite gel  SRP + herbal gel  Chlosite gel  Herbal gel | Gels application 3 times: after the SRP, and 2 times more with an interval of 10 days | Significant improvement of clinical parameters for SRP + chlosite gel and SRP + herbal gel compared with SRP. |
| Lecic et al., 2016 (32) | RCT  3 months | 15; 21-52; 8/7 | PI  BI  PPD  CAL | Chronic periodontitis with at least two bilateral periodontal sites probing ≥5 mm | SRP  SRP + chlorhexidine solution  SRP + chlorhexidine gel  SRP + chlorhexidine chip | - SRP + chlorhexidine solution : after SRP, once a day for 5 days.  - SRP + chlorhexidine gel: once a day for 5 days.  - SRP + chlorhexidine chip: one after the SRP | Except for chlorhexidine chip after 3 months for BI and PPD, no other parameters were found to be significantly improved. |
| Mamajiwala et al., 2019 (34) | RCT  6 months | 45; 35.8 ± 3.11 (18-55); 22/23 | PI  GI  mSBI  PPD  CAL | Mild to moderate periodontitis. PPD > 5 mm post initial therapy | SRP + placebo gel  SRP + boric acid (0.75%) gel  SRP + chlorhexidine (1%) gel | One gel application after the SRP | Significant improvement of clinical parameters for all test groups compared to control group, but no differences between test groups. |

**Supplemental table 3 - Periodontal parameters evaluation at baseline and after treatment in chlorhexidine studies**

| **Study** | **Treatments** | | **Clinical parameters at baseline** | **Clinical parameters at the end of the treatment** | **Differences between treatment modality** |
| --- | --- | --- | --- | --- | --- |
| Oosterwaal et al., 1991 (23)  36 weeks | SRP + placebo or a chlorhexidine gel (2%) or a stannous fluoride gel (4%) or amine fluoride gel (1.25%) | | Mean PPD: 7.6 + 0.7 mm  Mean BOP: 1.0 + 0 | Mean PPD: 4.7 +1.0 mm  Mean BOP: 0.2 + 0.4 | No significant differences between all tested procedures at 12 and 36 weeks. |
| Unsal et al., 1994 (33)  3 months | SRP  SRP + chlorhexidine (1%) gel  SRP + tetracycline paste | | PI:  SRP: 1.36 ± 0.39  SRP + C: 1.49 ± 0.44  GI:  SRP: 1.06 ± 0.23  SRP + C: 1.04 ± 0.25  BI:  SRP: 48.92 ± 15.19  SRP + C: 45.95 ± 12.82  PPD:  SRP: 5.14 ± 1.45  SRP + C: 4.90 ± 1.11  PAL:  SRP: 3.66 ± 1.22  SRP + C: 4.03 ± 1.50 | PI:  SRP: 0.60 ± 0.33  SRP + C: 0.54 ± 0.37  GI:  SRP: 0.55 ± 0.17  SRP + C: 0.42 ± 0.19  BI:  SRP: 10.12 ± 2.52  SRP + C: 5.86 ± 3.63  PPD change:  SRP: 1.83 ± 0.54  SRP + C: 1.58 ± 0.96  PAL change:  SRP: 1.04 ± 0.16  SRP + C: 0.70 ± 1.09 | No significant differences between groups were found. |
| Vinholis et al., 2001 (24)  3 months | SRP  SRP + irrigation with collagen gel  SRP + irrigation with chlorhexidine collagen-hamsana (1%) gel | | PI:  SRP: 2.40  SRP + C: 2.25  SRP + CC: 2.20  GI:  SRP: 2.00  SRP + C: 1.75  SRP + CC: 1.67  GR:  SRP: 0.50  SRP + C: 0.62  SRP + CC: 0.60  PPD:  SRP: 5.80  SRP + C: 5.75  SRP + CC: 5.53  CAL:  SRP: 6.30  SRP + C: 6.45  SRP + CC: 6.07 | PI:  SRP: 1.20  SRP + C: 1.25  SRP + CC: 0.93  GI:  SRP: 1.10  SRP + C: 1.25  SRP + CC: 0.90  GR:  SRP: 0.75  SRP + C: 0.83  SRP + CC: 0.93  PPD:  SRP: 2.70  SRP + C: 2.17  SRP + CC: 2.17  CAL:  SRP: 3.45  SRP + C: 3.00  SRP + CC: 3.10 | No significant differences between groups for PI, PPD, CAL, GI. The group SRP + CC demonstrated a GR mean higher than the other groups. |
| Gupta et al., 2008 (25)  3 months | SRP  SRP + doxycycline hyclate (10%) gel  SRP + xanthan based chlorhexidine (1.5%) gel | | PPD:  SRP: 6.02 ± 1.15  SRP + DH: 6.16 ± 1.17  SRP + CHX: 6.40 ± 0.89  CAL:  SRP: 6.00 ± 1.11  SRP + DH: 6.06 ± 1.14  SRP + CHX: 6.53 ± 1.00 | Mean PPD reduction:  SRP: 1.73 ± 0.94  SRP + DH: 2.75 ± 1.33  SRP + CHX: 2.76 ± 1.25  Mean CAL gain:  SRP: 0.86 ± 0.68  SRP + DH: 1.73 ± 0.90  SRP + CHX: 2.03 ± 1.12 | Significant PPD reduction for SRP + DH compared to SRP alone. Significant CAL gain for test groups compared to SRP alone. |
| Paolantonio et al., 2009 (26)  6 months | SRP  SRP + xanthan-chlorhexidine digluconate/dihydroxychloride (2.5%) gel | | BOP (%):  SRP: 100  SRP +C: 33.7 | 3 months:  PPD:  Difference between SRP and SRP +C: 0.87  CAL:  Difference between SRP and SRP +C: 0.94  BOP (%):  SRP: 45.9  SRP +C:33.7  6 months:  PPD:  Difference between SRP and SRP +C: 0.83  CAL:  Difference between SRP and SRP +C: 0.90  BOP (%):  SRP: 55.1  SRP +C: 38.8 | No differences in terms of PI and mGI  SRP + C induced improved results in terms of PPD reduction and CAL gain vs SRP alone at 3 and 6 months  No statistical difference between groups for BOP |
| Chandra et al., 2011 (27)  3 months | SRP  SRP + chlosite  Chlosite | | PI: non-smoker  SRP: 1.2  SRP + C:1.3  C:1.2  PI: smoker  SRP:1.3  SRP + C: 1.8  C: 1.4  RAL: non-smoker  SRP: 9.7  SRP + C: 10.8  C:10.3  RAL: smoker  SRP: 9.7  SRP + C: 10.5  C: 9.8  BI: non-smoker  SRP: 0.8  SRP + C: 0.4  C: 0.5  BI: smoker  SRP: 0.4  SRP + C: 0.5  C: 0.5  GI: non-smoker  SRP: 1.8  SRP + C: 1.4  C: 1.3  GI: smoker  SRP: 1.3  SRP + C: 1.1  C: 1.2 | PI: non-smoker  SRP: 0.2  SRP + C: 0.2  C: 0.5  PI: smoker  SRP: 0.7  SRP + C: 0.6  C: 1.2  RAL: non-smoker  SRP: 7.3  SRP + C: 8.8  C: 8.2  RAL: smoker  SRP: 7.4  SRP + C: 8.3  C: 7.8  BI: non-smoker  SRP: 0  SRP + C: 0  C: 0.1  BI: smoker  SRP: 0  SRP + C: 0  C: 0  GI: non-smoker  SRP: 0.2  SRP + C: 0.2  C: 0.2  GI: smoker  SRP: 0.2  SRP + C: 0.4  C: 0.4 | For non-smokers:  Mean RAL gain:  SRP: 24.7%  SRP + CHL: 18.5%  CHL: 20.4%  In smokers:  SRP: 23.7%  SRP + CHL: 21%  CHL: 20.4%  Significant compared to baseline values. No statistical differences between smokers and non-smokers. |
| Chauhan et al., 2013 (28)  3 months | SRP  SRP + hyaluronan gel  SRP + chlorhexidine (1.5%) gel | PI:  SRP: 2.85 ± 0.10  SRP + H: 2.87 ± 0.10  SRP + C: 2.89 ± 0.11  GI:  SRP: 2.82 ± 0.07  SRP + H: 2.84 ± 0.08  SRP + C: 2.82 ± 0.07  PPD:  SRP: 5.90 ± 0.27  SRP + H: 5.93 ± 0.60  SRP + C: 5.95 ± 0.31  CAL:  SRP: 6.10 ± 0.38  SRP + H: 6.13 ± 0.54  SRP + C: 6.15 ± 0.36 | | Mean PI reduction:  SRP: 2.19 ± 0.17  SRP + H: 2.23 ± 0.18  SRP + C: 2.23 ± 0.16  Mean GI reduction:  SRP: 2.16 ± 0.22  SRP + H: 2.22 ± 0.19  SRP + C: 2.15 ± 0.21  Mean PPD reduction:  SRP: 1.60 ± 0.27  SRP + H: 2.50 ± 0.42  SRP + C: 2.48 ± 0.32  Mean CAL gain:  SRP: 0.55 ± 0.16  SRP + H: 1.25 ± 0.20  SRP + C: 1.13 ± 0.27 | Significant difference between SRP + hyaluronan gel / SRP + chlorhexidine gel and SRP for PPD and CAL. |
| Chitsazi et al., 2013 (29)  3 months | SRP  SRP + xanthan-based chlorhexidine gel (CHX) | PPD:  SRP: 4.9 + 0.78  SRP + CHX: 5.05 + 0.75  CAL:  SRP: 3.9 + 0.58  SRP + CHX: 4.15 + 0.67  REC:  SRP: -0.85 + 0.56  SRP + CHX: -1.075 + 1.01  BOP (%):  SRP: 90 + 12.56  SRP + CHX: 95 + 10.25 | | PPD:  SRP: 3.25 + 0.65  SRP + CHX: 3.75 + 0.79  CAL:  SRP: 3.4 + 0.6  SRP + CHX: 3.67 + 0.65  REC:  SRP: 0.3 + 1.22  SRP + CHX: 0.27 + 0.49  BOP (%):  SRP: 21.25 + 9.1  SRP + CHX: 5 + 10.2 | Improvements were significant for all parameters in both groups. Only Bop was statistically different between groups at 3 months (*p*=0.001). |
| Jain et al., 2013 (31)  6 months | SRP  SRP + xanthan based chlorhexidine (1.5%) gel | PPD:  SRP: 5.2 + 0.48  SRP + CHX: 5.20 + 0.48  CAL:  SRP: 11.43 + 2.7  SRP+CHX: 11.70 + 2.8  GI:  SRP: 1.77+0.56  SRP + CHX: 1.8 + 0.61  PI:  SRP: 1.77 + 0.43  SRP + CHX: 1.03 + 0.49  BOP (%):  SRP: 1.87 + 0.9  SRP + CHX: 1.73 + 0.78 | | PPD:  SRP: 3.07 + 0.69  SRP + CHX: 2.5 + 0.73  CAL:  SRP: 9.2 + 2.8  SRP + CHX: 10.03 + 2.9  GI:  SRP: 0.77 + 0.67  SRP + CHX: 0.57 + 0.62  PI:  SRP: 1.03 + 0.49  SRP + CHX: 1.03 + 0.49  BOP (%):  SRP: 0.47 + 0.62  SRP + CHX: 0.2 + 0.48 | Significant intergroup differences were found at 3 months for PPD, CAL and BOP. |
| Phogat et al., 2014 (30)  3 months | SRP  SRP + chlosite gel (1.5%)  SRP + herbal gel  Chlosite gel (1.5%)  Herbal gel |  | | Mean changes at 3 months  PI:  SRP: 1.134 ± 0.144  SRP + C: 2.245 ± 0.191  SRP + H: 1.785 ± 0.001  GI:  SRP: 1.231 ± 0.001  SRP + C: 2.281 ± 0.212  SRP + H: 1.864 ± 0.105  PPD:  SRP: 2.264 ± 0.031  SRP + C: 3.764 ± 0.010  SRP + H: 2.917 ± 0.082  CAL:  SRP: 2.405 ± 0.079  SRP + C: 2.913 ± 0.051  SRP + H: 2.784 ±0.056 | Significant differences between SRP + chlosite gel/ SRP + herbal gel compared to SRP alone in term of PI, GI, PPD, and CAL (*p*<0.001). |
| Lecic et al., 2016 (32)  3 months | SRP  SRP + chlorhexidine solution (0.2%)(CHX solution)  SRP + chlorhexidine gel (0.5%) (CHX gel)  SRP + chlorhexidine chip | PI :  SRP: 1.38 ± 0.2  SRP + CHX gel: 1.30 ± 0.2  BI:  SRP: 1.80 ± 0.4  SRP + CHX gel: 1.70 ± 0.4  PPD:  SRP: 5.25 ± 0.5  SRP + CHX gel: 5.05 ± 1.0  CAL:  SRP: 4.05 ± 1.3  SRP + CHX gel: 3.75 ± 1.2 | | PI :  SRP: 0.41 ± 0.11  SRP + CHX gel: 0.41 ± 0.05  BI:  SRP: 0.70 ± 0.4  SRP + CHX gel: 0.40 ± 0.5  PPD:  SRP: 3.25 ± 0.5  SRP + CHX gel: 2.95 ± 0.7  CAL:  SRP: 3.20 ± 0.7  SRP + CHX gel: 3.40 ± 0.8 | At 3 months, no statistical difference between SRP alone and SRP + CHX gel (p<0.05). |
| Mamajiwala et al., 2019 (34)  6 months | SRP + placebo gel  SRP + boric acid (0.75%) gel  SRP + chlorhexidine (1%) gel | PI:  SRP + P: 1.63 ± 0.35  SRP + B: 1.56 ± 0.20  SRP + C: 1.42 ± 0.21  GI:  SRP + P: 1.28 ± 0.36  SRP + B: 1.21 ± 0.22  SRP + C: 1.34 ± 0.22  mSBI:  SRP + P: 1.50 ± 0.56  SRP + B: 1.52 ± 0.43  SRP + C: 1.49 ± 0.25  PPD:  SRP + P: 6.98 ± 0.97  SRP + B: 6.78 ± 1.48  SRP + C: 7.21 ± 1.12  CAL:  SRP + P: 2.21 ± 0.98  SRP + B: 2.15 ± 1.01  SRP + C: 2.32 ± 0.98 | | PI:  SRP + P: 1.31 ± 0.24  SRP + B: 1.11 ± 0.86  SRP + C: 1.02 ± 0.13  GI:  SRP + P: 0.98 ± 0.21  SRP + B: 0.74 ± 0.35  SRP + C: 0.69 ± 0.38  mSBI:  SRP + P: 1.05 ± 0.14  SRP + B: 0.76 ± 0.52  SRP + C: 0.71 ± 0.85  PPD:  SRP + P: 5.54 ± 1.01  SRP + B: 4.35 ± 0.62  SRP + C: 4.60 ± 0.89  CAL:  SRP + P: 1.98 ± 1.11  SRP + B: 1.21 ± 0.89  SRP + C: 1.32 ± 1.10  No 3 months data | Significant improvement of all the clinical parameter for test groups compared to control group (*p*<0.05), but not statistical differences between test groups. |

**Supplemental table 4 - Characteristics of the included studies evaluating the effect of gels loaded with metronidazole**

| **Study** | **Design; duration** | **Sample size ; mean age; gender (M/F)** | **Clinical parameters evaluated** | **Inclusion criteria** | **Treatment** | **Local administration** | **Findings** |
| --- | --- | --- | --- | --- | --- | --- | --- |
| Lie et al., 1998 (36) | RCT  3 months | 18; 36-77; NA | PPD  RAL (relative attachment level)  BOP | Moderate to severe periodontitis patient with no systemic diseases. Three interproximal sites at teeth with comparable root  anatomy were selected in each patient. Additional inclusion criteria were probing depths ≥ 5 mm combined with  bleeding on probing. | SRP  SRP + metronidazole gel (25%)  SRP + tetracycline gel (3%) | 2 treatment sessions (baseline + 1 week) | SRP alone appeared as effective as the drug  augmented regimens, although there was a weak but non-significant tendency for better  results in sites treated with the antibiotic drugs. Benefit of the microbiological outcome in test groups. Significant decrease of BOP at 3 months for test groups. |
| Kinane et al., 1999 (37) | RCT  6 months | 79 ; 45+/-6.4 ; 29/50 | PI  Modified GI  PPD  CAL  BOP | Periodontitis  Persistent pockets that did not respond favorably to SRP  4 sites with PPD > 5 mm + BOP | SRP  SRP + minocycline gel (2%)  SRP + metronidazole gel (25%) | Minocycline gel was applied at baseline and repeated 2 times at 2 and 4 weeks.  Metronidazole gel was repeated after 7 days | Both locally applied antimicrobials systems improved SRP outcomes. |
| Palmer et al., 1999 (39) | RCT  6 months | 84; 35-65; 43/47 | PPD  CAL (level)  BOP  Plaque score (presence or absence 4 sites on each tooth) | Moderate to advanced periodontitis | SRP  SRP + metronidazole systemic (200mg tid, 7 days)  SRP + metronidazole (25%) gel | SRP (partial mouth disinfection, in two sessions, T0 and one week after) +/- treatment at all sites more than 4 mm | There were no differences in any clinical measure in response to the three treatment regimens at 2 or 6 months for either smokers or non-smokers. Biologically, proportion reduction at 6 months of spirochaetes (less in smokers than in non-smokers). |
| Griffiths et al., 2000 (41) | RCT  9 months | 45; 46.5; 42/46 | PPD  CAL (level)  BOP | At least 2 sites in each quadrant with PPD > 4mm | SRP  SRP + metronidazole gel (25%) | Gel applied at baseline and 7 days after | The adjunctive use of gel induced an improvement in terms of PPD reduction, CAL gain vs SRP alone. |
| Stelzel et al., 2000 (38) | RCT  9 months | 59; 47 ;35/24 | PPD  CAL (level)  BOP | At least 2 pockets per quadrant with PPD > 4mm | SRP  SRP + metronidazole gel (25%) | Gel was applied after SRP and 7d after | Minor advantages obtained from the gel application vs SRP. |
| Akncbay et al., 2007 (40) | RCT  6 months | 15; 31-47 ( 38 ± 6); 7/8 | PI  GI  Gingival bleeding time index (GBTI)  PPD  CAL (loss)  GR | Moderate to severe chronic periodontitis.  PPD of 5–7 mm with BOP. At least 4 nonadjacent  sites of the single rooted teeth between the upper and lower  second premolars | SRP  SRP + vehicle gel (chitosan)  SRP + metronidazole + chitosan | Two gel application after the completion of SRP and 1 week after | The adjunctive use of gels improved PPD value vs SRP at 6 months. Significant difference between SRP + M vs SRP at 3 months and SRP + C at 6 months. |
| Pandit et al., 2013 (35) | RCT  3 months | 20; NA; 11/9 | GI  PI  PPD  CAL | At least three teeth with probing pocket depth of 5‑8 mm + BOP | SRP  SRP + minocycline microsphere gel  SRP + metronidazole gel | Minocycline microspheres gel application at day 1  Metronidazole gel application at day 1 and 7 | Treatment with minocycline and metronidazole gels improved PPD and CAL vs SRP alone. |

**Supplemental table 5 - Periodontal parameters evaluation at baseline and after treatment in metronidazole studies**

| **Study** | **Treatments** | **Clinical parameters at baseline** | **Clinical parameters at the end of the treatment** | **Differences between treatment modality** |
| --- | --- | --- | --- | --- |
| Lie et al., 1998 (36)  3 months | SRP  SRP + metronidazole (25%) gel  SRP + tetracycline (3%) gel | PPD:  Metronidazole: 5.0 (1.1)  Scaling: 5.1 (1.2)  Tetracycline: 5.2 (1.5)  RAL:  Metronidazole: 13.2 (1.2)  Scaling: 13.4 (1.8)  Tetracycline: 13.8 (2.5) | PPD:  Metronidazole: 3.4 (1.0)  Scaling: 4.0 (1.4)  Tetracycline: 3.5 (1.6)  RAL:  Metronidazole: 12.3 (1.5)  Scaling: 13.2 (1.5)  Tetracycline: 12.6 (2.0) | Weak but non-significant tendency for better results in sites treated with antibiotics drugs. Significative BOP reduction at 3 months. |
| Kinane et al., 1999 (37)  6 months | SRP  SRP + minocycline gel (2%) m  SRP + metronidazole gel (25%) M | PPD:  SRP: 5.48 + 0.17  SRP + m: 5.58 + 0.14  SRP + M: 5.50 + 0.15  MGI:  SRP: 2.18 + 0.1  SRP + m: 1.91 + 0.15  SRP + M: 2.13 + 0.11  BOP (%):  SRP: 92.5%  SRP + m: 89.22%  SRP + M: 88.58% | 3 months :  PPD reduction:  SRP: -0.87 + 0.15  SRP + m: -1.01 + 0.14  SRP + M: -0.91 + 0.21  CAL gain:  SRP: 0.54 + 0.15  SRP + m: 0.42 + 0.20  SRP + M: 0.54 + 0.16  MGI:  SRP: 1.56 + 0.08  SRP + m: 1.41 + 0.13  SRP + M: 1.57 + 0.09  BOP (%):  SRP: 56.25%  SRP + m: 44.05%  SRP + M: 46.89%  6 months :  PPD reduction:  SRP: -0.71 + 0.19  SRP + m: -1.10 + 0.16  SRP + M: -0.93 + 0.20  CAL gain:  SRP: 0.54 + 0.14  SRP + m: 0.57 + 0.11  SRP + M: 0.54 + 0.21  MGI:  SRP: 1.51 + 0.16  SRP + m: 1.50 + 0.12  SRP + M: 1.50 + 0.14  BOP (%):  SRP: 54.55%  SRP + m: 51.19%  SRP + M: 55.26% | No statistical data available for minocycline and metronidazole groups. |
| Palmer et al., 1999 (39)  6 months | SRP  SRP + metronidazole systemic (200mg tid, 7 days)  SRP + metronidazole (25%) gel | Plaque score: (smoker)  SRP: 18.2 (9.1)  SRP + M (S): 21.3 (14.6)  SRP + M (G): 18.7 (13.1)  Plaque score: (non- smoker)  SRP: 19.3 (14.8)  SRP + M (S): 22.1 (13.7)  SRP + M (G): 21.8 (14.5)  BOP (%): (smoker)  SRP: 21.4 (9.4)  SRP + M (S): 20.2 (12.2)  SRP + M (G): 24.2 (13.6)  BOP (%): (non-smoker)  SRP : 24.2 (18.7)  SRP + M (S): 28.7 (16.7)  SRP + M (G): 28.0 (16.5)  PPD : (smoker)  SRP : 5.73 (0.51)  SRP + M (S): 5.84 (0.43)  SRP + M (G): 5.69 (0.42)  PPD : (non-smoker)  SRP : 5.92 (0.49)  SRP + M (S): 6.10 (0.57)  SRP + M (G): 6.04 (0.75) | Plaque score: (smoker)  SRP: 11.2 (7.8)  SRP + M (S): 13.8 (16.3)  SRP + M (G): 15.2 (12.1)  Plaque score: (non-smoker)  SRP: 13.1 (10.9)  SRP + M (S): 13.6 (8.2)  SRP + M (G): 13.6 (8.9)  BOP (%): (smoker)  SRP: 12.1 (6.9)  SRP + M (S): 9.7 (5.1)  SRP + M (G): 13.6 (12.2)  BOP (%): (non-smoker)  SRP : 18.2 (16.3)  SRP + M (S): 16.1 (10.8)  SRP + M (G): 15.1 (7.5)  PPD : (smoker)  SRP : 4.61 (0.70)  SRP + M (S): 4.64 (0.61)  SRP + M (G): 4.38 (0.69)  PPD : (non-smoker)  SRP : 3.96 (0.37)  SRP + M (S): 4.27 (0.55)  SRP + M (G): 4.11 (0.63) | No differences in any clinical measure in response to the three treatment regimens at 6 months for either smokers or non-smokers. |
| Griffiths et al., 2000 (41)  9 months | SRP  SRP + metronidazole gel (25%) | BOP (%):  SRP: 68%  SRP+ M: 70%  PPD:  SRP: 6 + 0.5  SRP + M: 5.9 + 0.6 | BOP (%):  SRP: 42%  SRP+ M: 35%  Not provided | Significant differences for PPD and CAL gain between groups at 3 months in favor of SRP + M:  PPD: 0.4mm  CAL: 0.3mm |
| Stelzel et al., 2000 (38)  9 months | SRP  SRP + metronidazole gel (25%) | PPD:  SRP: 5.99 + 0.69  SRP + M: 6.17 + 0.74  CAL:  SRP: 6.32 + 1.22  SRP + M: 6.43 + 1.11  BOP (%):  SRP: 69 + 22  SRP + M: 73 + 20 | PPD:  SRP: 5.04 + 0.92  SRP + M: 4.76 + 0.68  CAL:  SRP: 5.83 + 0.98  SRP + M: 5.69 + 0.97  BOP (%):  SRP: 45 + 33  SRP + M: 37 + 27 | Significant differences between SRP + gel and SRP for PPD and BOP changes. |
| Akncbay et al., 2007 (40)  6 months | SRP  SRP + vehicle gel (chitosan)  SRP + metronidazole + chitosan | Not provided | Not provided | PPD reduction:  SRP: 0.94mm  SRP + vehicle gel: 1.21 mm  SRP + metronidazole + chitosan: 1.48 mm |
| Pandit et al., 2013 (35)  3 months | SRP  SRP + minocycline microsphere gel  SRP + metronidazole (25%) gel | PPD:  SRP: 6.85 + 0.81  SRP + minocycline: 6.8 + 1  SRP + metronidazole: 6.25 + 0.91  CAL:  SRP: 6.65 + 1.75  SRP + minocycline: 7.05 + 1.65  SRP + metronidazole: 6.6 + 1.99 | PPD:  SRP: 4.6 + 0.82  SRP + minocycline: 3.75 + 0.8  SRP + metronidazole: 4.1 + 0.91  CAL:  SRP: 4.95 + 1.65  SRP + minocycline: 4.6 + 1.76  SRP + metronidazole: 4.95 + 1.65 | Significant difference between SRP and SRP + minocycline for PPD and CAL. |

**Supplemental table 6 – Characteristics of the included studies evaluating the effect of gels loaded with satranidazole**

| **Study** | **Design; duration** | **Sample size ; mean age; gender (M/F)** | **Clinical parameters evaluated** | **Inclusion criteria** | **Treatment** | **Local administration** | **Findings** |
| --- | --- | --- | --- | --- | --- | --- | --- |
| Priyanka et al., 2015 (42) | RCT  6 months | 70; 30-50; 37/33 | PI  GI  PPD  CAL | PPD ≥ 5mm and/or CAL ≥ 4mm and vertical bone loss ≥3mm | SRP + placebo  SRP + satranidazole (3%) gel | One application after SRP | Satranidazole application improved SRP outcomes. |
| Priyanka et al., 2017 (43) | RCT  6 months | 60; 30-50; 60/0 | PI  GI  PPD  CAL | Chronic periodontitis. PPD ≥ 5 mm or clinical attachment level CAL ≥ 4 to 6 mm and vertical bone loss ≥ 3 mm | SRP + placebo gel  SRP + satranidazole (3%/0.1 mL) gel | One gel application after the SRP | Significant improvement of PPD and CAL for test group compared to control group. |

**Supplemental table 7 - Periodontal parameters evaluation at baseline and after treatment in satranidazole studies**

| **Study** | **Treatments** | **Clinical parameters at baseline** | **Clinical parameters at the end of the treatment** | **Differences between treatment modality** |
| --- | --- | --- | --- | --- |
| Priyanka et al., 2015 (42)  6 months | SRP + placebo  SRP + satranidazole (3%) (S) | PPD:  SRP + P: 7.41 + 1.27  SRP + S: 7.21 + 1.49  CAL:  SRP + P: 8.12 + 1.02  SRP + S: 7.92 + 1.24  PI:  SRP + P: 2.81 ± 0.19  SRP + S: 2.80 ± 0.18  GI:  SRP + P: 2.59 ± 0.20  SRP + S: 2.61 ± 0.25 | 3 months:  Mean PPD reduction:  SRP + P: 1.23 ± 0.53  SRP + S: 2.15 ± 1.24  Mean CAL gain:  SRP + P: 0.63 ± 1.15  SRP + S: 2.27 ± 1.62  PI:  SRP + P: 2.63 ± 0.18  SRP + S: 2.62 ± 0.17  GI:  SRP + P: 1.89 ± 0.38  SRP + S: 1.72 ± 0.36  6 months:  Mean PPD reduction:  SRP + P: 1.49 ± 1.01  SRP + S: 4.10 ± 1.11  Mean CAL:  SRP + P: 1.13 ± 0.49  SRP + S: 4.20 ± 1.12  PI:  SRP + P: 2.76 ± 0.21  SRP + S: 2.72 ± 0.18  GI:  SRP + P: 1.73 ± 0.37  SRP + S: 1.22 ± 0.28 | At 3 and 6 months PPD decrease and CAL gain were more significantly important in SRP + S group (*p*=0.001) while no significant changes were observed for PI and GI. |
| Priyanka et al., 2017 (43)  6 months | SRP + placebo gel  SRP + satranidazole (3%/0.1 mL) gel (S) | PI:  SRP + P: 2.88 ± 0.45  SRP + S: 2.86 ± 0.22  GI:  SRP + P: 2.72 ± 0.41  SRP + S: 2.77 ± 0.27  PPD:  SRP + P: 8.99 ± 1.44  SRP + S: 8.78 ± 1.38  CAL:  SRP + P: 7.57 ± 1.51  SRP + S: 7.92 ± 1.34 | 3 months:  PI:  SRP + P: 2.59 ± 0.16  SRP + S: 2.45 ± 0.34  GI:  SRP + P: 1.99 ± 0.32  SRP + S: 1.67 ± 0.14  Mean PPD reduction:  SRP + P: 1.68 ± 0.23  SRP + S: 2.37 ± 0.14  Mean CAL gain:  SRP + P: 1.64 ± 0.05  SRP + S: 2.11 ± 0.21  6 months:  PI:  SRP + P: 2.54 ± 0.35  SRP + S: 2.50 ± 0.23  GI:  SRP + P: 1.75 ± 0.15  SRP + S: 1.42 ± 0.11  Mean PPD reduction:  SRP + P: 1.97 ± 0.22  SRP + S: 3.05 ± 0.22  Mean CAL gain:  SRP + P: 1.88 ± 0.21  SRP + S: 2.89 ± 0.14 | Significant improvement for PPD and CAL at 6 months (*p*=0.001) and for GI, PPD, CAL at 3 months for test group compared to control group. |

**Supplemental table 8 - Characteristics of the included studies evaluating the effect of gels loaded with tetracyclines**

| **Study** | **Design; duration** | **Sample size ; mean age; gender (M/F)** | **Clinical parameters evaluated** | **Inclusion criteria** | **Treatment** | **Local administration** | **Findings** |
| --- | --- | --- | --- | --- | --- | --- | --- |
| Jeong et al., 1994 (49) | RCT  3 months | 16; 28-58; 6/10 | Plaque index  Sulcus bleeding index  PPD  CAL | At least 1 single rooted tooth that exhibited probing depth of 4 to 6 mm in each jaw quadrant | SRP  Tetracycline (5%) containing gel alone  SRP + tetracycline (5%) containing gel  SRP + tetracycline (5%) + citric acid (33%) containing gel | 1 application at baseline | The use of a mixture of tetracycline + citric acid improves SRP outcomes. |
| Graça et al., 1997 (47) | RCT  3 months | 26; 38.1; 6/20 | Presence or absence of marginal plaque  Marginal gingival bleeding  PPD  BOP  CAL | Presence of a minimum of 2 true pockets 5-10 mm on separate teeth with attachment loss > 4 mm  Radiographic evidence of alveolar bone loss, bleeding on deep probing, and/or suppuration at the study site | SRP  SRP + minocycline (2%) gel | 3 applications (baseline + 2 weeks + 4 weeks) | Adjunctive minocycline improved SRP outcomes in terms of CAL gain and reduction of BOP. |
| Lie et al., 1998 (36) | RCT  3 months | 18; 36-77; NA | BOP  PPD  RAL (relative attachment level) | Moderate to severe periodontitis patient with no systemic diseases. Three interproximal sites at teeth with comparable root  anatomy were selected in each patient. Additional inclusion criteria were probing depths ≥5 mm combined with  bleeding on probing. | SRP  SRP + metronidazole gel (25%)  SRP + tetracycline gel (3%) | 2 treatment sessions (baseline + 1 week) | SRP alone appeared as effective as the drug  augmented regimens, although there was a weak but non-significant tendency for better  results in sites treated with the antibiotic drugs. Benefit of the microbiological outcome in test groups. Significant decrease of BOP at 3 months for test groups. |
| Kinane et al., 1999 (37) | RCT  6 months | 79 ; 45+/-6.4 ; 29/50 | PI  Modified GI  PPD  CAL  BOP | Periodontitis  Persistent pockets that did not respond favorably to SRP  4 sites with PPD > 5 mm + BOP | SRP  SRP + minocycline gel (2%)  SRP + metronidazole gel (25%) | Minocycline gel was applied at baseline and repeated 2 times at 2 and 4 weeks.  Metronidazole gel was repeated after 7 days | Both locally applied antimicrobials systems improved SRP outcomes. |
| Eickholz et al., 2002 (45) | RCT  6 months | 111; 49.9; 41/70 | PPD  Relative attachment level (RAL)  GI  PI | At least 23 yo  Plaque control record < 35%  At least 3 single rooted teeth with PPD > 4mm + BOP or PPD > 5mm | SRP  SRP + vehicle gel  SRP + doxycycline gel (PLGA) (15%) | Gel was applied at baseline | The adjunctive use of gel improved RAL-V gain and PPD reduction vs SRP. |
| Gupta et al., 2008 (25) | RCT  3 months | 30; 25-75; NA | PI  GI  PPD  CAL  GM (gingival margin) | Untreated moderate to severe periodontitis or recurrent periodontitis without periodontal surgery at least for the last 24 months. A minimum of three teeth, distributed in the mouth to represent isolated experimental units (i.e., at least one tooth apart), with probing pocket depth (PPD) of 5 mm to 8 mm that bled on probing at the initial visit | SRP  SRP + doxycycline hyclate (10%) gel  SRP + xanthan based chlorhexidine (1.5%) gel | One gel application after the completion of SRP | Adjunctive use of both gels led to improved PPD compared to SRP alone. |
| Srirangarajan et al., 2011 (50) | RCT  9 months | 50; 35-55; 27/23 | PI  GI  PPD  RAL | Moderate to severe chronic periodontitis.  good systemic  health, ≥ 1 sites with a PPD of 4 to 6 mm | SRP  SRP + doxycycline microspheres  SRP + doxycycline gel | Drug application right after the SRP | Improvement of all clinical and biological parameters in all groups. |
| Jain et al., 2012 (48) | RCT  9 months | 15; NA; NA | BI  PPD  RAL | Moderate and severe chronic periodontitis.  CAL loss of 4 mm on at least two teeth (for moderate) and CAL loss > 5 mm on at least two teeth | SRP  SRP + minocycline (2%) gel | Gel application after the SRP, at 2 and 4 weeks after the first gel application | PPD values significantly less than control group at 6 and 9 months. Improvement of biological parameters at 9 months compared to control group. |
| Ahamed et al., 2013 (44) | RCT  6 months | 12; 25-55; 7/5 | PI  BI  GI  PPD  CAL | Chronic periodontitis. ≥ 5 nonadjacent periodontal pockets measuring ≥ 5 mm. | SRP  SRP + doxycycline hyclate (1%) gel | Gel application after the SRP | Better clinical parameters in test group compared to control group. |
| Pandit et al., 2013 (35) | RCT  3 months | 20; NA; 11/9 | PI  GI  PPD  CAL | At least three teeth with probing pocket depth of 5‑8 mm + BOP | SRP  SRP + minocycline microsphere gel  SRP + metronidazole gel | Minocycline microspheres gel application at day 1  Metronidazole gel application at day 1 and 7 | Treatment with minocycline and metronidazole gels improved PPD and CAL vs SRP alone. |
| Madi et al., 2018 (51) | RCT  3 months | 45; 20-45; NA | PI  GI  PPD  CAL | Moderate chronic periodontitis. PPD 4–6 mm in at least 3 permanent teeth | SRP + placebo (chitosan)  SRP + nano-doxycycline (1%)  SRP + doxycycline (1%) | Gel application after the SRP | Significant improvement of PPD and CAL for nano-doxycycline group compared to the other groups at 3 months. Significant improvement of CAL for doxycycline group compared to placebo group at 3 months. |

**Supplemental table 9 - Periodontal parameters evaluation at baseline and after treatment in tetracyclines studies**

| **Study** | **Treatments** | **Clinical parameters at baseline** | **Clinical parameters at the end of the treatment** | **Differences between treatment modality** |
| --- | --- | --- | --- | --- |
| Jeong et al., 1994 (49)  3 months | SRP  Tetracycline (5%) containing gel alone  SRP + tetracycline (T) (5%) containing gel  SRP + tetracycline (5%) + citric acid (C) (33%) containing gel | PI:  SRP: 1.47 + 0.13  SRP + T: 1.50 + 0.13  SRP + TC: 1.4 + 0.16  Gingival sulcus bleeding index:  SRP: 2.53 + 0.52  SRP + T: 2.63 + 0.47  SRP + TC: 2.6 + 0.5  PPD:  SRP: 4.67 + 0.62  SRP + T: 4.94 + 0.73  SRP + TC: 5.07 + 0.7  CAL:  SRP: 5.07 + 1.03  SRP +T: 5.06 + 0.93  SRP + TC: 5.27 + 0.73 | PI:  SRP: 0.53 + 0.13  SRP + T: 0.5 + 0.16  SRP + TC: 0.47 + 0.17  Gingival sulcus bleeding index:  SRP: 1.13+0.83  SRP + T: 0.81 + 0.66  SRP + TC: 0.87 + 0.83  PPD:  SRP: 3 + 0.65  SRP + T: 3 + 0.73  SRP + TC: 2.47 + 0.74  CAL:  SRP: 3.46 + 0.74  SRP +T: 3.31 + 1.01  SRP + TC: 2.93 + 0.96 | Improvement for all parameters from baseline in all groups.  PPD: SRP + TC gives better outcomes than SRP alone compared to the other groups (*p*<0.05) at 12 weeks. |
| Graça et al., 1997 (47)  3 months | SRP  SRP + minocycline (M) (2%) gel | Plaque (%):  SRP + placebo gel: 6.31 (3.47)  SRP + M: 6.38 (2.84)  BOP (%):  SRP + placebo gel: 9.92 (4.05)  SRP + M: 8.92 (3.12)  PPD:  SRP + placebo gel: 5.74 (0.35)  SRP + M: 5.93 (0.48)  CAL:  SRP + placebo gel: 6.83 (0.73)  SRP + M: 6.86 (1.04) | Plaque (%):  SRP + placebo gel: 3.08 (2.6)  SRP + M: 3.15 (3.31)  BOP (%):  SRP + placebo gel: 2.69 (0.95)  SRP + M: 2.08 (1.66)  PPD:  SRP + placebo gel: 3.44 (0.47)  SRP + M: 3.29 (0.49)  CAL:  SRP + placebo gel: 5.27 (0.76)  SRP + M: 4.91 (0.72) | Improvement for all parameters from baseline.  BOP and CAL gain: SRP + M gives significant better outcomes than SRP + placebo (*p*<0.05). |
| Lie et al., 1998 (36)  3 months | SRP  SRP + metronidazole (25%) gel  SRP + tetracycline (3%) gel | PPD:  Metronidazole: 5.0 (1.1)  Scaling: 5.1 (1.2)  Tetracycline: 5.2 (1.5)  RAL:  Metronidazole: 13.2 (1.2)  Scaling: 13.4 (1.8)  Tetracycline: 13.8 (2.5) | PPD:  Metronidazole: 3.4 (1.0)  Scaling: 4.0 (1.4)  Tetracycline: 3.5 (1.6)  RAL:  Metronidazole: 12.3 (1.5)  Scaling: 13.2 (1.5)  Tetracycline: 12.6 (2.0) | Weak but non-significant tendency for better results in sites treated with antibiotics drugs. Significative BOP reduction at 3 months. |
| Kinane et al., 1999 (37)  6 months | SRP  SRP + minocycline gel (2%) m  SRP + metronidazole gel (25%) M | PPD:  SRP: 5.48 + 0.17  SRP + m: 5.58 + 0.14  SRP + M: 5.50 + 0.15  MGI:  SRP: 2.18 + 0.1  SRP + m: 1.91 + 0.15  SRP + M: 2.13 + 0.11  BOP (%):  SRP: 92.5%  SRP + m: 89.22%  SRP + M: 88.58% | 3 months :  Mean PPD reduction:  SRP: -0.87 + 0.15  SRP + m: -1.01 + 0.14  SRP + M: -0.91 + 0.21  Mean CAL gain:  SRP: 0.54 + 0.15  SRP + m: 0.42 + 0.20  SRP + M: 0.54 + 0.16  MGI:  SRP: 1.56 + 0.08  SRP + m: 1.41 + 0.13  SRP + M: 1.57 + 0.09  BOP (%):  SRP: 56.25%  SRP + m: 44.05%  SRP + M: 46.89%  6 months :  Mean PPD reduction:  SRP: -0.71 + 0.19  SRP + m: -1.10 + 0.16  SRP + M: -0.93 + 0.20  Mean CAL gain:  SRP: 0.54 + 0.14  SRP + m: 0.57 + 0.11  SRP + M: 0.54 + 0.21  MGI:  SRP: 1.51 + 0.16  SRP + m: 1.50 + 0.12  SRP + M: 1.50 + 0.14  BOP (%):  SRP: 54.55%  SRP + m: 51.19%  SRP + M: 55.26% | No statistical data available for minocycline and metronidazole groups. |
| Eickholz et al., 2002 (45)  6 months | SRP  SRP + vehicle gel  SRP + doxycycline gel (PLGA) (15%) | PPD:  SRP: 7.36 + 1.65  SRP + V: 7.33 + 1.57  SRP + D: 7.33 + 1.55  RAL:  SRP: 9.16 + 1.98  SRP + V: 9.13 + 2.17  SRP + D: 9.15 + 1.99  PI:  SRP: 0.55 + 0.75  SRP + V: 0.5 + 0.78  SRP + D: 0.57 + 0.8  GI:  SRP: 1.81 + 0.7  SRP + V: 1.88 + 0.69  SRP + D: 1.81 + 0.55  BOP (%):  SRP: 79  SRP + V: 85  SRP + D: 84 | Mean PPD reduction:  SRP: -2.5+1.6  SRP + V: -2.6 + 1.5  SRP + D: -2.9 + 1.3  Mean RAL gain:  SRP: 1.8 + 1.7  SRP + V: 1.7 + 1.9  SRP + D: 2 + 1.6  PI reduction:  SRP: -0.2 + 1  SRP + V: -0.2 + 1  SRP + D: -0.2 + 1  GI reduction:  SRP: -0.8 + 1.1  SRP + V: -1 + 1.2  SRP + D: -1.1 + 1  BOP (%) reduction:  SRP: -40  SRP + V: -50  SRP + D: -59 | PPD and GI reduction were significantly improved in SRP + D group at 3 months. |
| Gupta et al., 2008 (25)  3 months | SRP  SRP + doxycycline hyclate (10%) gel  SRP + xanthan based chlorhexidine (1.5%) gel | PPD:  SRP: 6.02 ± 1.15  SRP + DH: 6.16 ± 1.17  SRP + CHX: 6.40 ± 0.89  CAL:  SRP: 6.00 ± 1.11  SRP + DH: 6.06 ± 1.14  SRP + CHX: 6.53 ± 1.00 | Mean PPD reduction:  SRP: 1.73 ± 0.94  SRP + DH: 2.75 ± 1.33  SRP + CHX: 2.76 ± 1.25  Mean CAL reduction:  SRP: 0.86 ± 0.68  SRP + DH: 1.73 ± 0.90  SRP + CHX: 2.03 ± 1.12 | Significant PPD reduction for SRP + DH compared to SRP alone. Significant CAL gain for test groups compared to SRP alone. |
| Srirangarajan et al., 2011 (50)  9 months | SRP  SRP + doxycycline microspheres (60%)  SRP + doxycycline gel (10%) | PPD:  SRP : 4.96 ± 0.56  SRP + M: 5.16 ± 0.32  SRP + G: 5.15 ± 0.29  RAL:  SRP : 9.17 ± 0.57  SRP + M: 9.11 ± 0.45  SRP + G: 9.12 ± 0.45 | 3 months:  PPD:  SRP : 4.74 ± 0.54  SRP + M: 4.65 ± 0.35  SRP + G: 4.82 ± 0.33  RAL:  SRP : 8.60 ± 0.57  SRP + M: 7.84 ± 0.22  SRP + G: 8.83 ± 0.29  9 months:  PPD:  SRP : 4.92 ± 0.52  SRP + M: 4.69 ± 0.34  SRP + G: 4.99 ± 0.35  RAL:  SRP : 8.81 ± 0.65  SRP + M: 8.62 ± 0.42  SRP + G: 7.93 ± 0.82 | PPD reduction :  SRP: 0.04mm  SRP + M: 0.45 ± 0.02 mm  SRP + G: 0.16mm  RAL gain:  SRP: 0.36 mm  SRP + M: 0.49 mm  SRP + G: 1.19 mm  No intergroup comparison. |
| Jain et al., 2012 (48)  9 months | SRP  SRP + minocycline (M) (2%) gel | BI:  SRP : 0.727  SRP + M: 0.863  PPD:  SRP: 6.02 + 0,7  SRP + M: 6.17 + 0,9  RAL:  SRP: 12.91  SRP + M : 13.03 | 3 months:  BI:  SRP: 0.545  SRP + M: 0.318  PPD:  SRP: 4.22 + 0,94  SRP + M: 3.71 + 0,97  RAL:  SRP: 11.40  SRP + M : 11.30  9 months:  BI:  SRP: 0.611  SRP + M: 0.500  PPD:  SRP: 4.63  SRP + M: 3.81  RAL:  SRP: 11.29  SRP + M : 11.16 | PPD values at 6 (3.64 ± 0.83 mm) and 9 months (3.81 ± 0.79 mm) were significantly improved compared to control group (4.24 ± 0.95 mm at 6 and 4.63 ± 0.94 mm at 9 months). Improvement of biological parameters in test group compared to control group. |
| Ahamed et al., 2013 (44)  6 months | SRP  SRP + doxycycline hyclate (1%) gel | GI:  SRP: 1.55  SRP + D: 1.49  PPD:  SRP: 6.6 ± 0.3  SRP + D: 6.4 ± 0.2 | Not provided | Significant improvement of BOP, PPD and CAL gain at 6 months for experimental group compared to control group. |
| Pandit et al., 2013 (35)  3 months | SRP  SRP + minocycline microsphere gel  SRP + metronidazole (25%) gel | PPD:  SRP: 6.85 + 0.81  SRP + minocycline: 6.8 + 1  SRP + metronidazole: 6.25 + 0.91  CAL:  SRP: 6.65 + 1.75  SRP + minocycline: 7.05 + 1.65  SRP +metronidazole: 6.6 + 1.99 | PPD:  SRP: 4.6 + 0.82  SRP + minocycline: 3.75 + 0.8  SRP + metronidazole: 4.1 + 0.91  CAL:  SRP: 4.95 + 1.65  SRP + minocycline: 4.6 + 1.76  SRP + metronidazole: 4.95 + 1.65 | Significant difference between SRP and SRP + minocycline for PPD and CAL. |
| Madi et al., 2018 (51)  3 months | SRP + placebo (chitosan)  SRP + doxycycline (DOX) (1%)  SRP + nano-doxycycline (nDOX) (1%) | PI:  SRP + P: 0.84 ± 0.21  SRP + DOX: 0.83 ± 0.20  SRP + nDOX: 0.83 ± 0.12  GI:  SRP + P: 2.49 ± 0.30  SRP + DOX: 2.44 ± 0.36  SRP + nDOX: 2.43 ± 0.56  PPD:  SRP + P: 5.31 ± 0.67  SRP + DOX: 5.33 ± 0.67  SRP + nDOX: 5.21 ± 1.25  CAL:  SRP + P: 3.83 ± 0.42  SRP + DOX: 3.73 ± 0.53  SRP + nDOX: 3.82 ± 0.42 | PI:  SRP + P: 0.73 ± 0.12  SRP + DOX: 0.68 ± 0.07  SRP + nDOX: 0.68 ± 0.08  GI:  SRP + P: 0.91 ± 0.22  SRP + DOX: 0.73 ± 0.13  SRP + nDOX: 0.55 ± 0.17  PPD:  SRP + P: 4.40 ± 0.84  SRP + DOX: 4.10 ± 0.54  SRP + nDOX: 3.90 ± 0.34  CAL:  SRP + P: 3.80 ±0.41  SRP + DOX: 3.30 ± 0.51  SRP + nDOX: 2.80 ± 0.32 | PPD and CAL significantly improved in nDOX group compared to the others at 3 months (p<0.01). Only CAL significantly improved in DOX group compared to placebo group at 3 months (p<0.01). |

**Supplemental table 10 - Characteristics of the included studies evaluating the effect of gels loaded with macrolides**

| **Study** | **Design; duration** | **Sample size ; mean age; gender (M/F)** | **Clinical parameters evaluated** | **Inclusion criteria** | **Treatment** | **Local administration** | **Findings** |
| --- | --- | --- | --- | --- | --- | --- | --- |
| Pradeep et al., 2008 (52) | RCT  3 months | 80; 25-50; 46/34 | Modified gingival index (mGI)  Modified sulcus bleeding index (mSBI)  PPD  CAL | Chronic periodontitis.  At least one pocket  with PPD ≥ 5 mm and BOP | SRP  SRP + azithromycin (0.5%) gel | One gel application after the completion of SRP | Adjunctive use of gel did only improved both biological the clinical parameters. |
| Agarwal et al., 2012 (53) | RCT  6 months | 61; 30-50; 61/0 | PI  GI  mSBI  PPD  CAL | Chronic periodontitis.  PPD ≥5mm, CAL ≥ 4 to 6mm and vertical bone loss ≥ 3mm | SRP + placebo gel  SRP + clarithromycin (0.5%) gel | Gel application after the SRP | Enhanced clinical outcomes after 6 months for SRP + CLM compared to SRP. |
| Pradeep, Bajaj et al., 2013 (54) | RCT  9 months | 63; 30-50; 63/0 | PI  mSBI  PPD  CAL | Chronic periodontitis.  PPD ≥ 5mm or CAL ≥ 4 to 6mm and vertical bone loss ≥ 3mm | SRP + placebo  SRP + azithromycin (0.5%) gel | Gel application after the SRP | Clinical parameters significantly improved in test group compared to control group at 3, 6 and 9 months (except for mSBI). |
| Pejčić et al., 2015 (55) | RCT  6 months | 50; 47.6;27/23 | PPD  CAL  BOP  PI | At least one pocket, from 5 up to 7 mm in depth | SRP  SRP + clindamycin (10mg in 500mg) gel | One application after SRP | Clindamycin gel enhanced SRP outcomes through reduction of PPD and CAL gain. |

**Supplemental table 11 - Periodontal parameters evaluation at baseline and after treatment in macrolides studies**

| **Study** | **Treatments** | **Clinical parameters at baseline** | **Clinical parameters at the end of the treatment** | **Differences between treatment modality** |
| --- | --- | --- | --- | --- |
| Pradeep et al., 2008 (52)  3 months | SRP  SRP + azithromycin (0.5%) gel (A) | MGI:  SRP: 2.22 ± 0.40  SRP + A : 2.24 ± 0.43  mSBI :  SRP: 1.54 ± 0.39  SRP + A : 1.81 ± 0.41  PPD :  SRP: 6.53 ± 1.19  SRP + A : 6.53 ± 0.99  CAL :  SRP: 5.53 ± 1.06  SRP + A : 5.33 ± 1.11 | MGI:  SRP: 0.68 ± 0.14  SRP + A : 1.00 ± 0.58  mSBI :  SRP: 0.62 ± 0.14  SRP + A : 0.74 ± 0.45  Mean PPD reduction:  SRP: 2.13 ± 0.35  SRP + A : 2.53 ± 0.52  Mean CAL gain:  SRP: 0.60 ± 0.63  SRP + A : 1.07 ± 0.70 | Clinical results significantly enhanced for SRP + A compared to SRP. |
| Agarwal et al., 2012 (53)  6 months | SRP + placebo gel  SRP + clarithromycin (0.5%) gel | GI:  SRP + P: 1.83 ± 0.38  SRP + CLM: 1.80 ± 0.33  PI:  SRP + P: 4.37 ± 0.66  SRP + CLM: 4.29 ± 0.77  mSBI:  SRP + P: 2.20 ± 0.49  SRP + CLM: 2.26 ± 0.48  PPD:  SRP + P: 7.17 ± 0.72  SRP + CLM: 7.17 ± 0.62  CAL:  SRP + P: 6.37 ± 0.47  SRP + CLM: 6.42 ± 0.71 | 3 months :  GI:  SRP + P: 1.41 ± 0.33  SRP + CLM: 1.32 ± 0.25  PI:  SRP + P: 3.32 ± 0.65  SRP + CLM: 3.15 ± 0.67  mSBI:  SRP + P: 1.55 ± 0.27  SRP + CLM: 1.42 ± 0.18  PPD:  SRP + P: 5.92 ± 0.87  SRP + CLM: 4.89 ± 0.46  CAL:  SRP + P: 5.26 ± 0.42  SRP + CLM: 4.71 ± 0.49  6 months :  GI:  SRP + P: 1.38 ± 0.41  SRP + CLM: 1.06 ± 0.28  PI:  SRP + P: 3.22 ± 0.57  SRP + CLM: 2.82 ± 0.64  mSBI:  SRP + P: 1.44 ± 0.27  SRP + CLM: 1.36 ± 0.24  PPD:  SRP + P: 6.07 ± 0.88  SRP + CLM: 4.64 ± 0.63  CAL:  SRP + P: 5.69 ± 0.46  SRP + CLM: 4.90 ± 0.46 | SRP + CLM patients demonstrated significant enhanced clinical outcomes (PI, GI, PPD, CAL) compared to control group (*p*<0.05). |
| Pradeep, Bajaj et al., 2013 (54)  9 months | SRP + placebo gel  SRP + azithromycin (0.5%) gel | PI:  SRP + G: 4.52 ± 0.51  SRP + A: 4.59 ± 0.50  mSBI:  SRP + G: 2.22 ± 0.50  SRP + A: 2.32 ± 0.46  PPD:  SRP + G: 7.56 ± 0.93  SRP + A: 7.70 ± 0.82  CAL:  SRP + G: 6.48 ± 0.51  SRP + A: 6.63 ± 0.49 | 3 months:  PI:  SRP + G: 3.78 ± 0.61  SRP + A: 3.55 ± 0.51  mSBI:  SRP + G: 1.73 ± 0.37  SRP + A: 1.65 ± 0.15  PPD:  SRP + G: 6.44 ± 0.85  SRP + A: 5.92 ± 0.62  CAL:  SRP + G: 6.11 ± 0.51  SRP + A: 4.85 ± 0.36  9 months:  PI:  SRP + G: 3.74 ± 0.53  SRP + A: 2.66 ± 0.48  mSBI:  SRP + G: 1.47 ± 0.29  SRP + A: 1.38 ± 0.17  PPD:  SRP + G: 6.92 ± 0.73  SRP + A: 5.44 ± 0.52  CAL:  SRP + G: 6.30 ± 0.54  SRP + A: 4.19 ± 0.39 | Mean change from baseline between control and experimental groups:  PI: 6.21 (*p*<0.001*)  mSBI : 1.52 (*p*= 0.136)  PPD : 5.51 (*p*<0.001*)  CAL : 12.55 (*p*<0.001*)  All significant except mSBI.  Same results at 3 months (all significantly improved except mSBI). |
| Pejčić et al., 2015 (55)  6 months | SRP  SRP + clindamycin (2%) gel (C) | PPD:  SRP: 5.92 + 0.67  SRP + C: 6.04 + 0.68  CAL:  SRP: 1.62 + 0.29  SRP + C: 1.58 + 0.32 | 3 months:  PPD:  SRP: 5.45 + 0.47  SRP + C: 4.76 + 0.65  CAL:  SRP: 1.08 + 0.39  SRP + C: 0.94 + 0.31  6 months:  PPD:  SRP: 5.56 + 0.65  SRP + C: 3.62 + 0.31  CAL:  SRP: 0.98 + 0.28  SRP + C: 0.60 + 0.44 | PPD and CAL were significantly improved in test group (*p*<0.001) at 6 months (only PPD at 3 months). |

**Supplemental table 12 - Characteristics of the included studies evaluating the effect of gels loaded with fluoroquinolone**

| **Study** | **Design; duration** | **Sample size ; mean age; gender (M/F)** | **Clinical parameters evaluated** | **Inclusion criteria** | **Treatment** | **Local administration** | **Findings** |
| --- | --- | --- | --- | --- | --- | --- | --- |
| Flemmig et al., 2011 (56) | RCT  3 months | 57; 18-75; 24/33 | PPD  GR  CAL  BOP  Suppuration  PI  FI | Moderate to severe chronic periodontitis.  ≥ 12 teeth remaining. probing depth (PPD) of ≥ 5.4 mm at ≥ 4 teeth without radiographic signs of apical periodontitis | SRP + placebo gel  SRP + moxifloxacin (0.125%) gel  SRP + moxifloxacin (0.4%) gel  SRP + moxifloxacin (1.25%) gel | Gel application right after the SRP | No linear trend for PPD reduction with increasing moxifloxacin concentrations was found. SRP + MOX 0.4% leaded to better PPD reduction. *PG* reduced in all test groups. |
| Kadadasu et al., 2020 (57) | RCT  3 months | 20; >18; NA | PI  GI  PPD  CAL | Moderate‑to‑severe chronic generalized periodontitis with PPD > 5 mm and < 8 mm | SRP  SRP + moxifloxacin and ibuprofen | One gel application after the SRP | Clinical parameters significantly improved in test group compared to SRP alone. |

**Supplemental table 13 - Periodontal parameters evaluation at baseline and after treatment in fluoroquinolone studies**

| **Study** | **Treatments** | **Clinical parameters at baseline** | **Clinical parameters at the end of the treatment** | **Differences between treatment modality** |
| --- | --- | --- | --- | --- |
| Flemmig et al., 2011 (56)  3 months | SRP + placebo gel  SRP + moxifloxacin (0.125%) gel  SRP + moxifloxacin (0.4%) gel  SRP + moxifloxacin (1.25%) gel | PPD:  SRP + P: 6.4 ± 0.7  SRP + MOX 0.125: 6.8 ± 0.7  SRP + MOX 0.4: 6.5 ± 0.7 SRP + MOX 1.25: 6.3 ± 0.5  GR:  SRP + P: 0.5 ± 0.4  SRP + MOX 0.125: 0.5 ± 0.4  SRP + MOX 0.4: 0.6 ± 0.5 SRP + MOX 1.25: 0.5 ± 0.4  CAL:  SRP + P: 7.2 ± 1.1  SRP + MOX 0.125: 7.2 ± 0.9  SRP + MOX 0.4: 7.1 ± 0.8 SRP + MOX 1.25: 6.8 ± 0.9  BOP (%):  SRP + P: 73.2 ± 24.2  SRP + MOX 0.125: 71.7 ± 25.3  SRP + MOX 0.4: 71.4 ± 26.7 SRP + MOX 1.25: 74.8 ± 18.1  Suppuration (%):  SRP + P: 2.2 ± 5.9  SRP + MOX 0.125: 4.4 ± 13.1  SRP + MOX 0.4: 1.1 ± 4.3 SRP + MOX 1.25: 5.2 ± 12.3  PI (%):  SRP + P: 64.8 ± 34.7  SRP + MOX 0.125: 70.4 ± 26  SRP + MOX 0.4: 61.1 ± 31.9  SRP + MOX 1.25: 63.5 ± 21.9  FI (class):  SRP + P: 0.8 (0.7)  SRP + MOX 0.125: 1.1 ± 1  SRP + MOX 0.4: 0.7 ± 0.7  SRP + MOX 1.25: 0.8 ± 0.6 | Reduction:  PPD:  SRP + P: 1.0 ± 0.6  SRP + MOX 0.125: 1.1 ± 1.1  SRP + MOX 0.4: 1.5 ± 0.6 SRP + MOX 1.25: 1.2 ± 0.4  GR:  SRP + P: 0.4 ± 0.3  SRP + MOX 0.125: 0.4 ± 0.3  SRP + MOX 0.4: 0.7 ± 0.4 SRP + MOX 1.25: 0.4 ± 0.3  CAL:  SRP + P: -0.6 ± 0.5  SRP + MOX 0.125: -0.7 ± 0.9  SRP + MOX 0.4: -0.8 ± 0.4  SRP + MOX 1.25: -0.8 ± 0.4  BOP (%):  SRP + P: -2.9 ± 22.8  SRP + MOX 0.125: -0.1 ± 30.8  SRP + MOX 0.4: -12.1 ± 25.3 SRP + MOX 1.25: -18.6 ± 32.3  Suppuration (%):  SRP + P: -1.1 ± 4.3  SRP + MOX 0.125: -2.8 ± 14.4  SRP + MOX 0.4: -1.1 ± 4.3  SRP + MOX 1.25: -5.2 ± 12.3  PI (%):  SRP + P: -13.2 ± 25.7  SRP + MOX 0.125: -17 ± 47  SRP + MOX 0.4: 2.2 ± 27.7  SRP + MOX 1.25: -2.2 ± 24.5  FI (class):  SRP + P: 0 ± 0  SRP + MOX 0.125: -0.1 ± 0.4  SRP + MOX 0.4: 0 ± 0.4  SRP + MOX 1.25: 0 ± 0.4 | Significant superiority of  MOX 0.4 (*p*=0.023) in term of PPD reduction compared to the control group (PPD reduction of ≥ 1.5mm in this group). No correlation between MOX doses and PPD change.  *PG* reduced in all test groups. |
| Kadadasu et al., 2020 (57)  3 months | SRP  SRP + moxifloxacin and ibuprofen | PPD:  SRP: 6.08 ± 0.57  SRP + M/I: 6.03 ± 0.62  CAL:  SRP: 4.65 ± 0.65  SRP + M/I: 4.65 ± 0.65 | Mean PPD reduction:  SRP: 1.83 ± 0.47  SRP + M/I: 2.85 ± 0.33  Mean CAL gain:  SRP: 1.95 ± 0.22  SRP + M/I: 2.85 ± 0.29 | All clinical parameters significantly improved in test group compared to SRP alone (*p*<0.05). |

**Supplemental table 14 - Characteristics of the included studies evaluating the effect of gels loaded with statins**

| **Study** | **Design; duration** | **Sample size ; mean age; gender (M/F)** | **Clinical parameters evaluated** | **Inclusion criteria** | **Treatment** | **Local administration** | **Findings** |
| --- | --- | --- | --- | --- | --- | --- | --- |
| Pradeep & Thorat, 2010 (67) | RCT  6 months | 60; 25-45 (30.5 ± 4.1) ; 33/31 | mSBI  PI  PPD  CAL | Chronic periodontitis. Moderate (PPD from 5 to 6mm or CAL from 4 to 6 mm) or deep pockets (PPD≥ 7 mm or CAL of 6 to 9 mm) and vertical bone loss ≥ 3 mm | SRP + placebo  SRP + simvastatin (SMV) gel | Gel application right after the SRP | Greater decrease in gingival index and PPD and more CAL gain with significant infra-bony defect fill at sites treated with SRP + SMV. |
| Pradeep, Kumari, et al., 2013 (62) | RCT  9 months | 67; 30-50; 35/32 | mSBI  PI  PPD  CAL | Severe chronic periodontitis. PPD≥ 5 mm, CAL ≥ 4 mm and vertical bone loss ≥ 3 mm | SRP + placebo  SRP + atorvastatin (ATV) (1.2%) gel | Gel application after the SRP | At 3, 6 and 9 months greater clinical parameters (PPD and CAL). Greater bone fill found in ATV group. |
| Rao, Pradeep, Bajaj, et al., 2013 (64) | RCT  9 months | 40; 30-50; 40/0 | PI  mSBI  PPD  CAL  IBD depth | Chronic periodontitis with sites having PPD ≥ 5 mm or CAL ≥ 4 mm and vertical bone loss ≥ 3 mm  Smoker (> 10 cig/day) | SRP + simvastatin (1.2%) gel (SMV)  SRP + placebo | Gel application after the SRP | Greater decrease in mSBI and PPD and more CAL gain at sites treated with SRP + SMV in smokers with chronic periodontitis. |
| Pradeep et al., 2015 (61) | RCT  6 months | 70; 25-55; 33/37 | mSBI  PI  PPD  CAL | Chronic periodontitis. Moderate (probing depth (PPD) of 5-6 mm or clinical attachment level (CAL) of 4-6 mm or deep pockets (PPD ≥ 7 mm or CAL of 6 - 9 mm) and vertical bone loss ≥ 3 mm | SRP + placebo gel  SRP + rosuvastatin (1.2%) gel | Gel application right after the SRP | Significant improvement of sMBI, PPD, CAL and IBD in test group compared to control group. |
| S. Agarwal et al., 2016 (58) | RCT  6 months | 30; 25-50; 14/16 | PI  GI  PPD  CAL | Chronic periodontitis. PPD ≥ 5 mm and vertical bone loss ≥ 2 mm between the base of defect and adjacent alveolar crest | SRP + placebo gel  SRP + simvastatin (1.2mg) gel | Gel application after the SRP | PPD and CAL significantly improved in test group compared to control group. |
| Pradeep, Garg, et al., 2016 (69) | RCT  9 months | 90; 25-45;45/45 | PI  mSBI  PPD  CAL | Chronic periodontitis with PPD ≥ 5 mm, CAL ≥ 3 mm and angular bone loss ≥ 3 mm | SRP + placebo  SRP + rosuvastatin (1.2%) gel  SRP + atorvastatin (1.2%) gel | One application after SRP | Adjunctive use of 1.2% rosuvastatin gel results in significantly greater improvements than placebo and atorvastatin gels. |
| Grover et al., 2016 (66) | RCT  3 months | 50; 25-45; NA | PI  mSBI  PPD  RAL | Chronic periodontitis. PPD of 5–8 mm in posterior | SRP  SRP + simvastatin (1.2mg) gel | One application after SRP | Significant improvement of all parameters for simvastatin group compared to SRP, except for RAL. |
| Kumari et al., 2017 (59) | RCT  9 months | 71; 30-50; NA | mSBI  PPD CAL | Chronic periodontitis.  PPD ≥ 5 mm or CAL ≥ 4 mm and vertical bone loss ≥ 3 mm | SRP + placebo gel  SRP + atorvastatin (1.2%) gel | One application after SRP | Adjunctive use of 1.2% atorvastatin leads to better clinical and radiological parameters at 3 months. |
| Pradeep, Kanoriya, et al., 2017 (60) | RCT  9 months | 104; 30-50; 53/51 | mSBI  PI  PPD  CAL | Chronic generalized periodontitis.  PPD ≥ 5 mm or CAL ≥ 4 to 6 mm and vertical bone loss ≥ 3 mm | SRP + placebo  SRP + alendronate (1%) gel  SRP + atorvastatin (1.2%) gel | One application after SRP | PPD, CAL and defect depth reduction (DDR) significantly improved in test groups than in control groups at 3 months. |
| Pankaj et al., 2018 (70) | RCT  12 months | 90; 25-45 (34.32 ±5.09)  ; 44/46 | PI  mSBI  PPD  CAL  IBD | Chronic periodontitis. PPD ≥ 5 mm and clinical attachment (CA) loss > 3 mm | SRP + placebo gel  SRP + rosuvastatin (1.2%) gel  SRP + metformin (1%) gel | Gels application after the SRP | Test groups presented significantly better clinical improvement compared to control group. |
| Chatterjee et al., 2019 (68) | RCT  6 months | 100; 30-60; 47/53 | PI  mSBI  PPD  CAL  IBD | Periodontitis with clinical attachment level (CAL) ≥ 3 mm, probing depth (PPD) ≥ 4 mm, and vertical bone loss ≥ 3 mm | SRP + placebo gel  SRP + rosuvastatin (1.2%) gel | One gel application after the SRP | Improvement of the clinical parameters for rosuvastatin gel group compared to placebo group. |
| Shirke et al., 2019 (65) | RCT  6 months | 20; 35.7 (30-45); 10/10 | PI  mSBI  PPD  CAL | Chronic periodontitis patient. PPD and clinical attachment level (CAL) ≥ 5 mm, along with the presence of at least one pair of similar intraosseous defect which was ≥ 3 mm deep | SRP + placebo gel  SRP + atorvastatin (1.2%) gel | One gel application after the SRP | Greater improvement and significantly better bone fill in atorvastatin group was found compared to placebo group. |
| Rahman et al., 2020 (63) | RCT  3 months | 15; 35-60; 8/7 | API  PBI  PPD  RAL | Grade A and Stage II periodontitis. Patients with < 0.25% of bone loss per years, heavy rates of biofilm deposition but slow rate of progression. PPD ≤ 5 mm, RAL (relative attachment level) ≤ 4 mm, horizontal bone loss, requiring non-surgical treatment | SRP  SRP + antimicrobial photodynamic therapy (aPDT)  SRP + simvastatin (1.2%) gel | One simvastatin gel and aPDT after SRP | No significant clinical differences between group at 3 months. |

**Supplemental table 15 - Periodontal parameters evaluation at baseline and after treatment in statins studies**

| **Study** | **Treatments** | **Clinical parameters at baseline** | **Clinical parameters at the end of the treatment** | **Differences between treatment modality** |
| --- | --- | --- | --- | --- |
| Pradeep & Thorat, 2010 (67)  6 months | SRP + placebo gel  SRP + simvastatin (1.2%) gel (SMV) | PPD:  SRP + P: 6.87 ± 1.61  SRP + SMV: 7.43 ± 1.59  CAL:  SRP + P: 6.20 ± 1.65  SRP + SMV: 6.23 ± 1.45  mSBI:  SRP + P: 2.92 ± 0.75  SRP + SMV: 3.12 ± 0.68 | Mean PPD reduction:  SRP + P: 1.20 ± 1.24  SRP + SMV: 4.26 ± 1.59  Mean CAL gain:  SRP + P: 1.63 ± 1.99  SRP + SMV: 4.36 ± 1.92  mSBI:  SRP + P: 2.42 ± 1.15  SRP + SMV: 0.79 ± 0.69  No 3 months results | Greater decrease of PPD, mSBI and greater amount of CAL and infra-bony defect (IBD) gain in group SRP + SMV. |
| Pradeep, Kumari, et al., 2013 (62)  9 months | SRP + placebo  SRP + atorvastatin (ATV) (1.2%) gel | PI:  SRP + P: 1.73 ± 0.16  SRP + ATV: 1.71 ± 0.13  mSBI:  SRP + P: 2.71 ± 0.21  SRP + ATV: 2.76 ± 0.19  PPD:  SRP + P: 7.73 ± 0.14  SRP + ATV: 7.77 ± 1.30  CAL:  SRP + P: 7.40 ± 1.22  SRP + ATV: 7.03 ± 1.43  IBD depth:  SRP + P: 4.75 ± 0.51  SRP + ATV: 4.79 ± 0.54 | 3 months:  PI:  SRP + P: 1.12 ± 0.32  SRP + ATV: 1.04 ± 0.19  mSBI:  SRP + P: 1.57 ± 0.16  SRP + ATV: 1.39 ± 0.24  Mean PPD reduction:  SRP + P: 1.36 ± 0.54  SRP + ATV: 3.40 ± 0.56  Mean CAL gain:  SRP + P: 1.86 ± 0.55  SRP + ATV: 2.70 ± 0.54  9 months:  PI:  SRP + P: 0.74 ± 0.26  SRP + ATV: 0.68 ± 0.31  mSBI:  SRP + P: 1.61 ± 0.16  SRP + ATV: 1.13 ± 0.23  Mean PPD reduction:  SRP + P: 1.46 ± 0.55  SRP + ATV: 3.70 ± 0.59  Mean CAL gain:  SRP + P: 2.26 ± 0.51  SRP + ATV: 4.46 ± 0.60  IBD depth: (6 months)  SRP + P: 4.62 ± 0.50  SRP + ATV: 3.15 ± 0.40  IBD depth: (9 months)  SRP + P: 4.67 ± 0.51  SRP + ATV: 3.09 ± 0.40 | 1.2% ATV significantly improved the clinical parameters (mSBI, PPD, CAL) and improved bone fill in adjunct to SRP compared to placebo gel alone at 3 and 9 months. |
| Rao, Pradeep, Bajaj, et al., 2013 (64)  9 months | SRP + simvastatin (1.2%) gel (SMV)  SRP + placebo | FMPS:  SRP: 1.85 ± 0.28  SRP + SMV: 1.89 ± 0.25  mSBI:  SRP: 2.01 ± 0.4  SRP + SMV: 2.08 ± 0.35  PPD:  SRP: 7.93 ± 1.12  SRP + SMV: 7.87 ± 0.9  CAL:  SRP: 6.13 ± 0.94  SRP + SMV: 6.2 ± 1.16 | 3 months:  FMPS:  SRP: 1.06 ± 0.31  SRP + SMV: 1.10 ± 0.22  mSBI:  SRP: 1.54 ± 0.35  SRP + SMV: 1.15 ± 0.35  Mean PPD reduction:  SRP: 1.23 ± 1.01  SRP + SMV: 2.30 ± 1.06  Mean CAL gain:  SRP: 1.00 ± 0.79  SRP + SMV: 2.13 ± 0.94  9 months:  FMPS:  SRP: 0.89 ± 0.22  SRP + SMV: 0.91 ± 0.23  mSBI:  SRP: 1.91 ± 0.28  SRP + SMV: 1.20 ± 0.18  Mean PPD reduction:  SRP: 1.57 ± 1.22  SRP + SMV: 3.83 ± 1.26  Mean CAL gain:  SRP: 1.47 ± 1.41  SRP + SMV: 3.63 ± 1.10 | Significant improvement for mSBI, PPD and CAL at 3 months. |
| Pradeep et al., 2015 (61)  6 months | SRP + placebo gel  SRP + rosuvastatin (1.2%) gel | mSBI :  SRP + P : 3.93 ± 0.58  SRP + R : 4.07 ± 0.46  PPD :  SRP + P : 7.05 ± 0.65  SRP + R : 7.08 ± 0.74  CAL :  SRP + P : 6.24 ± 0.49  SRP + R : 6.27 ± 0.63  IBD :  SRP + P : 4.59 ± 0.74  SRP + R : 4.59 ± 0.62 | 3 months :  mSBI :  SRP + P : 2.37 ± 0.25  SRP + R : 1.04 ± 0.28  Mean PPD reduction :  SRP + P : 2.27 ± 0.1  SRP + R : 3.31 ± 0.28  Mean CAL gain:  SRP + P : 1.68 ± 0.14  SRP + R : 2.92 ± 0.07  6 months :  mSBI :  SRP + P : 2.44 ± 0.25  SRP + R : 0.35 ± 0.22  Mean PPD reduction :  SRP + P : 1.31 ± 0.24  SRP + R : 4.04 ± 0.34  Mean CAL gain:  SRP + P : 1.40 ± 0.15  SRP + R : 4.20 ± 0.17  IBD :  SRP + P : 4.13 ± 0.72  SRP + R : 2.36 ± 0.30 | All parameters were significantly improved in SRP + R group compared to control (p<0.05). |
| S. Agarwal et al., 2016 (58)  6 months | SRP + placebo gel  SRP + simvastatin (1.2%) gel | PI:  SRP + P: 1.991 ± 0.379  SRP + S: 1.966 ± 0.358  GI:  SRP + P: 1.825 ± 0.247  SRP + S: 1.841 ± 0.258  PPD:  SRP + P: 6.000 ± 0.694  SRP + S: 6.400 ± 0.894  CAL:  SRP + P: 7.966 ± 0.764  SRP + S: 8.500 ± 0.900 | 3 months:  Mean PPD reduction:  SRP + P: 1.67 ± 0.48  SRP + S: 2.63 ± 0.61  Mean CAL gain:  SRP + P: 1.53 ± 0.51  SRP + S: 2.23 ± 0.57  6 months:  PI:  SRP + P: 0.006 ± 0.130  SRP + S: 0.025 ± 0.076  GI:  SRP + P: 0.375 ± 0.268  SRP + S: 0.141 ± 0.142  Mean PPD reduction:  SRP + P: 1.70 ± 0.47  SRP + S: 2.77 ± 0.68  Mean CAL gain:  SRP + P: 1.50 ± 0.51  SRP + S: 2.40 ± 0.56 | No intergroups comparison. |
| Pradeep, Garg, et al., 2016 (69)  9 months | SRP + placebo (P)  SRP + rosuvastatin (1.2%) gel (R)  SRP + atorvastatin (1.2%) gel (A) | PI:  SRP + P: 2.85 ± 0.36  SRP + R: 2.96 ± 0.19  SRP + A: 2.92 ± 0.26  mSBI:  SRP + P: 2.81 ± 0.39  SRP + R: 2.88 ± 0.32  SRP + A: 2.85 + 0.36  PPD:  SRP + P: 7.29 ± 0.6  SRP + R: 7.37 ± 0.62  SRP + A: 7.29 ± 0.66  CAL:  SRP + P: 5.29 ± 0.6  SRP + R: 5.37 ± 0.62  SRP + A: 5.29 ± 0.66  IBD:  SRP + P: 5.92 ± 0.26  SRP + R: 5.98 ± 0.35  SRP + A: 5.96 ± 0.19 | PI:  SRP + P: 1.36 ± 0.61  SRP + R: 0.51 ± 0.26  SRP + A: 0.70 ± 0.23  mSBI:  SRP + P: 1.28 ± 0.58  SRP + R: 0.49 ± 0.27  SRP + A: 0.67 ± 0.21  PPD:  SRP + P: 5.44 ± 0.89  SRP + R: 2.77 ± 0.42  SRP + A: 3.92 ± 0.87  CAL:  SRP + P: 3.44 ± 0.89  SRP + R: 1.07 ± 0.26  SRP + A: 1.96 ± 0.85  IBD:  SRP + P: 5.81 ± 0.62  SRP + R: 1.66 ± 0.48  SRP + A: 2.62 ± 1.14  No 3 months results | Significant improvement were observed for statin drugs groups for PI, mSBI, PPD and CAL. Result were significantly improved with rosuvastatin compared to atorvastatin for PI, PPD, CAL and infrabony defect depth reduction. |
| Grover et al., 2016 (66)  3 months | SRP  SRP + simvastatin (SMV) (1.2 mg) gel | No available data | PI (mean):  SRP: 23.92 ± 9.63  SRP + SMV: 37.42 ± 11.47  mSBI:  SRP: 22.26 ± 12.08  SRP + SMV: 37.45 ± 11.33  PPD:  SRP: 14.14 ± 12.61  SRP + SMV: 22.66 ± 9.05  RAL:  SRP: 23.40 ± 8.94  SRP + SMV: 25.56 ± 10.44 | Significant improvement of all clinical parameters except RAL at 3 months (p<0.01). |
| Kumari et al., 2017 (59)  9 months | SRP + placebo gel  SRP + atorvastatin (1.2%) gel | PI:  SRP + P: 1.70 ± 0.18  SRP + A: 1.69 ± 0.14  mSBI:  SRP + P: 2.67 ± 0.26  SRP + A: 2.68 ± 0.25  PPD:  SRP + P: 7.03 ± 1.38  SRP + A: 6.97 ± 1.44  CAL:  SRP + P: 6.94 ± 1.41  SRP + A: 6.90 ± 1.40  IBD:  SRP + P: 4.71 ± 0.52  SRP + A: 4.73 ± 0.54 | 3 months  PI:  SRP + P: 1.12 ± 0.32  SRP + A: 1.10 ± 0.23  mSBI:  SRP + P: 1.53 ± 0.18  SRP + A: 1.41 ± 0.27  Mean PPD reduction:  SRP + P: 0.88 ± 0.60  SRP + A: 1.54 ± 1.17  Mean CAL gain:  SRP + P: 1.48 ± 0.97  SRP + A: 2.27 ± 1.07  PI: 9 months  SRP + P: 0.74 ± 0.24  SRP + A: 0.69 ± 0.29  mSBI:  SRP + P: 1.55 ± 0.20  SRP + A: 1.16 ± 0.25  Mean PPD reduction:  SRP + P: 0.91 ± 1.07  SRP + A: 3.84 ± 0.83  Mean CAL gain:  SRP + P: 1.85 ± 1.28  SRP + A: 4.06 ± 1.64  IBD: 9 months  SRP + P: 4.56 ± 0.53  SRP + A: 3.20 ± 0.39 | Significant improvement for mSBI, PPD, CAL and IBD at 3 months (*p*<0.05).  Significant improvement for mSBI, PPD, CAL and IBD at 9 months (*p*<0.05). |
| Pradeep, Kanoriya, et al., 2017 (60)  9 months | SRP + placebo  SRP + alendronate (1%) gel  SRP + atorvastatin (1.2%) gel | PI:  SRP + P: 1.98 ± 0.334  SRP + AL: 1.95 ± 0.356  SRP + AT: 1.94 ± 0.318  mSBI:  SRP + P: 1.93 ± 0.63  SRP + AL: 2.10 ± 0.66  SRP + AT: 2.00 ± 0.64  PPD:  SRP + P: 6.76 ± 1.22  SRP + AL: 6.96 ± 1.12  SRP + AT: 6.56 ± 1.38  CAL:  SRP + P: 6.13 ± 1.13  SRP + AL: 5.93 ± 0.94  SRP + AT: 6.13 ± 0.89  IBD:  SRP + P: 5.17 ± 0.35  SRP + AL: 5.18 ± 0.65  SRP + AT: 5.46 ± 0.81 | 3 months  PI:  SRP + P: 1.26 ± 0.2  SRP + AL: 1.28 ± 0.225  SRP + AT: 1.29 ± 0.206  mSBI:  SRP + P: 1.43 ± 0.56  SRP + AL: 1.16 ± 0.64  SRP + AT: 1.16 ± 0.59  Mean PPD reduction:  SRP + P: 0.33 ± 0.47  SRP + AL: 1.53 ± 0.50  SRP + AT: 1.06 ± 0.63  Mean CAL gain:  SRP + P: 0.16 ± 0.37  SRP + AL: 1.06 ± 0.52  SRP + AT: 1.66 ± 0.60  9 months  PI:  SRP + P: 0.59 ± 0.092  SRP + AL: 0.54 ± 0.11  SRP + AT: 0.57 ± 0.11  mSBI:  SRP + P: 0.80 ± 0.66  SRP + AL: 0.43 ± 0.50  SRP + AT: 0.40 ± 0.49  Mean PPD reduction:  SRP + P: 1.50 ± 1.07  SRP + AL: 4.30 ± 1.39  SRP + AT: 3.53 ± 1.27  Mean CAL gain:  SRP + P: 1.50 ± 1.07  SRP + AL: 3.50 ± 1.16  SRP + AT: 4.26 ± 1.08  IBD:  SRP + P: 5.04 ± 0.38  SRP + AL: 2.79 ± 0.30  SRP + AT: 3.47 ± 0.70 | CAL and PPD significantly improved in test groups at 3, 6 and 9 months for test groups compared to control group (*p*<0.05).  Alendronate was found to be significantly better than atorvastatin in term of PPD and CAL improvement. |
| Pankaj et al., 2018 (70)  12 months | SRP + placebo gel  SRP + rosuvastatin (1.2%) gel  SRP + metformin (1%) gel | PI:  SRP + P: 1.98 ± 0.33  SRP + R: 1.94 ± 0.31  SRP + M: 1.96 ± 0.35  mSBI:  SRP + P: 1.96 ± 0.61  SRP + R: 2.03 ± 0.61  SRP + M: 2.10 ± 0.66  PPD:  SRP + P: 6.80 ± 1.21  SRP + R: 7.30 ± 1.17  SRP + M: 7.40 ± 1.10  CAL:  SRP + P: 6.06 ± 1.17  SRP + R: 6.23 ± 1.01  SRP + M: 6.20 ± 1.12  IBD :  SRP + P: 5.15 ± 0.35  SRP + R: 5.26 ± 0.87  SRP + M: 5.18 ± 0.65 | 6 months  PI:  SRP + P: 0.79 ± 0.13  SRP + R: 0.81 ± 0.11  SRP + M: 0.76 ± 0.12  mSBI:  SRP + P: 1.16 ± 0.59  SRP + R: 0.86 ± 0.73  SRP + M: 0.70 ± 0.65  Mean PPD reduction:  SRP + P: 1.03 ± 0.76  SRP + R: 2.66 ± 0.844  SRP + M: 3.10 ± 1.15  Mean CAL gain:  SRP + P: 1.23 ± 0.90  SRP + R: 2.77 ± 1.14  SRP + M: 3.67 ± 1.03  IBD :  SRP + P: 5.20 ± 0.01  SRP + R: 3.74 ± 0.42  SRP + M: 3.63 ± 0.29  12 months  PI:  SRP + P: 0.59 ± 0.09  SRP + R: 0.53 ± 0.11  SRP + M: 0.53 ± 0.09  mSBI:  SRP + P: 0.83 ± 0.64  SRP + R: 0.50 ± 0.50  SRP + M: 0.46 ± 0.57  Mean PPD reduction:  SRP + P: 1.53 ± 1.07  SRP + R: 3.73 ± 1.28  SRP + M: 4.66 ± 1.21  Mean CAL gain:  SRP + P: 1.47 ± 1.07  SRP + R: 3.90 ± 1.15  SRP + M: 1.17 ± 1.17  IBD :  SRP + P: 5.04 ± 0.38  SRP + R: 3.08 ± 0.31  SRP + M: 3.17 ± 0.24  No 3 months data | PPD, CAL and IBD significantly improved for test groups compared to control group at 6 and 12 months (*p*<0.001), but no differences between test groups. |
| Chatterjee et al., 2019 (68)  6 months | SRP + placebo gel  SRP + rosuvastatin (1.2%) gel | PI:  SRP + P: 1.96 ± 0.47  SRP + R: 1.83 ± 0.58  mSBI :  SRP + P: 3.82 ± 0.75  SRP + R: 3.83 ± 0.73  PPD:  SRP + P: 6.25 ± 1.59  SRP + R:6.29 ± 1.17  CAL:  SRP + P: 7.10 ± 1.40  SRP + R: 7.32 ± 1.51  IBD:  SRP + P: 6.22 ± 1.50  SRP + R: 6.50 ± 1.71 | PI:  SRP + P: 0.98 ± 0.47  SRP + R: 0.994 ± 0.47  mSBI :  SRP + P: 1.59 ± 0.64  SRP + R: 1.54 ± 0.62  PPD:  SRP + P: 4.02 ± 0.65  SRP + R: 3.44 ± 0.73  CAL:  SRP + P: 4.5 ± 0.90  SRP + R: 4.18 ± 0.96  IBD:  SRP + P: 6.02 ± 0.299  SRP + R: 5.23 ± 1.46  No 3 months results | Significant improvement for PPD, CAL and IBD (*p*<0.05) for rosuvastatin group compared to placebo group. |
| Shirke et al., 2019 (65)  6 months | SRP + placebo gel  SRP + atorvastatin (1.2%) gel | PPD:  SRP + P: 7.45 ± 0.51  SRP + A: 7.50 ± 0.51  CAL:  SRP + P: 7.80 ± 0.52  SRP + A: 7.90 ± 0.55 | 3 months  PPD:  SRP + P: 6.20 ± 0.69  SRP + A: 5.60 ± 0.59  CAL:  SRP + P: 6.50 ± 0.76  SRP + A: 5.80 ± 0.62  6 months  PPD:  SRP + P: 5.65 ± 0.58  SRP + A: 4.45 ± 0.51  CAL:  SRP + P: 5.90 ± 0.64  SRP + A: 4.55 ± 0.51 | Significant PPD and CAL improvement at 3 and 6 months for atorvastatin group compared to placebo group (*p*<0.0001). |
| Rahman et al., 2020 (63)  3 months | SRP  SRP + antimicrobial photodynamic therapy (aPDT)  SRP + simvastatin (1.2%) gel | PPD:  SRP: 5.82 (1.17)  SRP + aPDT: 5.91 (1.14)  SRP + S: 5.65 (0.52)  RAL:  SRP: 9.36 (1.43)  SRP + aPDT: 10.73 (2.24)  SRP + S: 9.91 (0.54) | PPD:  SRP: 4.64 (1.29)  SRP + aPDT: 4.09 (1.22)  SRP + S: 3.73 (0.91)  RAL:  SRP: 8.18 (1.60)  SRP + aPDT: 8.09 (2.26)  SRP + S: 7.21 (0.94) | No statistical differences between groups (*p*<0.05). |

**Supplemental table 16 - Characteristics of the included studies evaluating the effect of gels loaded with metformin**

| **Study** | **Design; duration** | **Sample size ; mean age; gender (M/F)** | **Clinical parameters evaluated** | **Inclusion criteria** | **Treatment** | **Local administration** | **Findings** |
| --- | --- | --- | --- | --- | --- | --- | --- |
| Pradeep, Rao, et al., 2013 (72) | RCT  6 months | 41; 30-50 (37.2 ± 2.6); 20/21 | mSBI  PI  PPD  CAL | Chronic periodontitis.  PPD≥ 5 mm, CAL ≥ 4 mm and vertical bone loss ≥ 3 mm | SRP + placebo gel  SRP + metformin (0.5%) gel  SRP + metformin (1%) gel  SRP + metformin (1.5%) gel | Gel application after the SRP | Enhanced clinical parameters (PPD, CAL) and demonstrated better IBD fill for 1% MF compared to control group. |
| Rao, Pradeep, Kumari, et al., 2013 (71) | RCT  6 months | 50; 30-50 (34.6 ± 1.4); 50/0 | mSBI  PI  PPD  CAL | Generalized chronic periodontitis. PPD≥ 5 mm, CAL ≥ 4 mm and vertical bone loss ≥ 3 mm | SRP + placebo  SRP + metformin (1%) gel | Gels application after the SRP | Greater PPD reduction, bone fill and CAL gain in test group compared to control. |
| Pradeep, Patnaik, et al., 2016 (73) | RCT  6 months | 65; 25-50 (32.4 ± 2.1); 38/27 | mSBI  PI  PPD  CAL | Chronic periodontitis.  PPD ≥5 mm, CAL ≥4 mm, and vertical bone loss ≥3 mm | SRP + placebo gel  SRP + metformin (1%) gel | Gel application after the SRP | Adjunctive injection of metformin 1% improved PPD, CAL, mSBI and IBD at 3 months. |
| Pradeep, Patnaik, et al., 2017 (74) | RCT  9 months | 64; 30-50; 34/30 | PI  mSBI  PPD  CAL | Chronic generalized periodontitis. PPD ≥ 5 mm, CAL ≥ 4 mm and vertical bone loss ≥ 3 mm | SRP + placebo gel  SRP + metformin (1%) gel | One gel application after the SRP | PPD and CAL significantly improved in metformin group compared to control group for initial PPD ≥ 5 mm and ≥ 7 mm. |
| Pankaj et al., 2018 (70) | RCT  12 months | 90; 25-45 (34.32 ±5.09)  ; 44/46 | PI  mSBI  PPD  CAL  IBD | Chronic periodontitis. PPD ≥5mm and clinical attachment (CA) loss >3mm | SRP + placebo gel  SRP + rosuvastatin (1.2%) gel  SRP + metformin (1%) gel | Gels application after the SRP | Test groups presented significantly better clinical improvement compared to control group. |
| Kurian et al., 2018 (75) | RCT  12 months | 105; 41; 54/51 | PI  mSBI  PPD  CAL | Moderate-to-severe chronic periodontitis. PPD ≥ 5 mm, CAL ≥ 3 mm, and vertical bone loss ≥ 3 mm | SRP + placebo gel  SRP + metformin (1%) gel  SRP + aloe vera gel | One gel application after the SRP | Greater clinical parameters in test groups compared to the control group. |

**Supplemental table 17 - Periodontal parameters evaluation at baseline and after treatment in metformin studies**

| **Study** | **Treatments** | **Clinical parameters at baseline** | **Clinical parameters at the end of the treatment** | **Differences between treatment modality** |
| --- | --- | --- | --- | --- |
| Pradeep, Rao, et al., 2013 (72)  6 months | SRP + placebo gel  SRP + metformin (0.5%) gel  SRP + metformin (1%) gel  SRP + metformin (1.5%) gel | PI:  SRP + P: 1.87 ± 0.28  SRP + MF (0.5%): 1.86 ± 0.26  SRP + MF (1%): 1.90 ± 0.26  SRP + MF (1.5%): 1.89 ± 0.25  mSBI:  SRP + P: 2.00 ± 0.39  SRP + MF (0.5%): 2.05 ± 0.36  SRP + MF (1%): 2.04 ± 0.32  SRP + MF (1.5%): 2.08 ± 0.35  PPD:  SRP + P: 8.03 ± 1.10  SRP + MF (0.5%): 8.00 ± 0.79  SRP + MF (1%): 8.13 ± 0.94  SRP + MF (1.5%): 8.16 ± 0.75  CAL:  SRP + P: 6.27 ± 0.79  SRP + MF (0.5%): 6.40 ± 0.86  SRP + MF (1%): 6.43 ± 0.86  SRP + MF (1.5%): 6.30 ± 0.79  IBD depth:  SRP + P: 4.92 ± 0.57  SRP + MF (0.5%): 4.89 ± 0.50  SRP + MF (1%): 4.91 ± 0.50  SRP + MF (1.5%): 4.85 ± 0.52 | 3 months:  PI:  SRP + P: 1.08 ± 0.31  SRP + MF (0.5%): 1.11 ± 0.22  SRP + MF (1%): 1.10 ± 0.22  SRP + MF (1.5%): 1.09 ± 0.22  mSBI:  SRP + P: 1.54 ± 0.35  SRP + MF (0.5%): 1.14 ± 0.32  SRP + MF (1%): 1.11 ± 0.29  SRP + MF (1.5%): 1.13 ± 0.31  Mean PPD reduction:  SRP + P: 1.50 ± 0.73  SRP + MF (0.5%): 1.97 ± 1.07  SRP + MF (1%): 2.90 ± 0.92  SRP + MF (1.5%): 2.80 ± 0.89  Mean CAL gain:  SRP + P: 0.97 ± 0.41  SRP + MF (0.5%): 1.57 ± 0.57  SRP + MF (1%): 2.53 ± 0.78  SRP + MF (1.5%): 2.36 ± 0.56  6 months  PI:  SRP + P: 0.73 ± 0.21  SRP + MF (0.5%): 0.69 ± 0.13  SRP + MF (1%): 0.68 ± 0.11  SRP + MF (1.5%): 0.70 ± 0.12  mSBI:  SRP + P: 1.63 ± 0.29  SRP + MF (0.5%): 0.89 ± 0.30  SRP + MF (1%): 0.87 ± 0.27  SRP + MF (1.5%): 0.88 ± 0.28  Mean PPD reduction:  SRP + P: 1.77 ± 0.77  SRP + MF (0.5%): 2.97 ± 0.93  SRP + MF (1%): 4.00 ± 1.05  SRP + MF (1.5%): 3.80 ± 1.03  Mean CAL gain:  SRP + P: 1.33 ± 0.66  SRP + MF (0.5%): 2.23 ± 0.73  SRP + MF (1%): 3.83 ± 0.95  SRP + MF (1.5%): 3.60 ± 0.81  IBD depth: (6 months)  SRP + P: 4.73 ± 0.45  SRP + MF (0.5%): 3.86 ± 0.46  SRP + MF (1%): 3.34 ± 0.61  SRP + MF (1.5%): 3.47 ± 0.58 | In term of PPD and CAL, except at 3 months for placebo VS 0.5% MF: significant improvement for all test groups compared to control one (*p*<0.05) at 3 and 6 months. mSBI significantly improved at 3 and 6 months for all test groups. IBD significantly improved at 6 months. |
| Rao, Pradeep, Kumari, et al., 2013 (71)  6 months | SRP + placebo  SRP + metformin (1%) gel | PI:  SRP + placebo: 1.76 ± 0.20  SRP + M: 1.81 ± 0.21  mSBI:  SRP + placebo: 2.15 ± 0.35  SRP + M: 2.11 ± 0.30  PPD:  SRP + placebo: 7.63 ± 0.81  SRP + M: 7.50 ± 0.51  CAL:  SRP + placebo: 6.43 ± 0.77  SRP + M: 2.11 ± 0.30  IBD:  SRP + placebo: 5.08 ± 0.44  SRP + M: 4.99 ± 0.41 | 3 months  PI:  SRP + placebo: 1.12 ± 0.29  SRP + M: 1.04 ± 0.19  mSBI:  SRP + placebo: 1.73 ± 0.23  SRP + M: 1.22 ± 0.28  Mean PPD reduction:  SRP + placebo: 0.73 ± 0.64  SRP + M: 2.01 ± 0.71  Mean CAL gain:  SRP + placebo: 0.87 ± 0.57  SRP + M: 2.37 ± 0.72  6 months  PI:  SRP + placebo: 0.84 ± 0.13  SRP + M: 0.82 ± 0.14  mSBI:  SRP + placebo: 1.50 ± 0.26  SRP + M: 0.86 ± 0.31  Mean PPD reduction:  SRP + placebo: 0.87 ± 0.94  SRP + M: 3.17 ± 0.75  Mean CAL gain:  SRP + placebo: 1.47 ± 0.78  SRP + M: 3.27 ± 0.79  IBD: 6 months  SRP + placebo: 4.86 ± 0.28  SRP + M: 3.67 ± 0.30 | Significant improvement for mSBI, PPD, CAL and IBD filling for test group compared to control group at 3 and 6 months. |
| Pradeep, Patnaik, et al., 2016 (73)  6 months | SRP + placebo gel  SRP + metformin (M) (1%) gel | FMPS:  SRP + P: 1.8233 ± 0.25  SRP + M: 1.8433 ±0.17  mSBI:  SRP + P: 2.00 ± 0.69  SRP + M: 2.03 ± 0.61  PPD:  SRP + P: 7.96 ± 1.09  SRP + M: 8.03 ± 0.76  CAL:  SRP + P: 6.13 ± 0.93  SRP + M: 6.23 ± 0.77  IBD:  SRP + P: 4.7 ± 0.50  SRP + M: 5.01 ± 0.66 | 3 months  FMPS:  SRP + P: 1.04 ± 0.30  SRP + M: 1.13 ± 0.17  mSBI:  SRP + P: 1.50 ± 0.77  SRP + M: 1.06 ± 0.69  Mean PPD reduction:  SRP + P: 1.16 ± 0.69  SRP + M: 2.86 ± 0.57  Mean CAL gain:  SRP + P: 0.76 ± 0.62  SRP + M: 2.93 ± 0.44  6 months:  FMPS:  SRP + P: 0.70 ± 0.15  SRP + M: 0.58 ± 0.14  mSBI:  SRP + P: 1.30 ± 0.83  SRP + M: 0.90 ± 0.71  Mean PPD reduction:  SRP + P: 2.00 ± 1.06  SRP + M: 3.96 ± 0.66  Mean CAL gain:  SRP + P: 1.43 ± 0.97  SRP + M: 4.06 ± 0.86  IBD: 6 months  SRP + P: 4.53 ± 0.41  SRP + M: 3.65 ± 0.52 | PPD, CAL and mSBI significantly improved in SRP + M group compared to SRP +P group (*p*<0.05) at 3 months. At 6 months: FMPS PPD, CAL and IBD significantly improved. |
| Pradeep, Patnaik, et al., 2017 (74)  9 months | SRP + placebo gel  SRP + metformin (1%) gel | PI:  SRP + P: 1.8 ± 0.26 (1.75-1.94)  SRP + M: 1.9 ± .018 (1.78-1.92)  mSBI:  SRP + P: 2.1 ± 0.69 (1.78-2.28)  SRP + M: 2.1 ± 0.61 (1.84-2.29)  PPD:  SRP + P: 6.7 ± 0.46  SRP + M: 6.6 ± 0.46  CAL:  SRP + P: 6.0 ± 0.14 (5.62-6.45)  SRP + M: 6.4 ± 0.91 (6.11-6.77)  IBD:  SRP + P: 4.8 ± 0.49 (4.62-4.98)  SRP + M: 5.02 ± 0.64 (4.79-5.25) | 3 months  PI:  SRP + P: 1.1 ± 0.28 (0.93-1.14)  SRP + M: 1.1 ± 0.17 (1.08-1.2)  mSBI:  SRP + P: 1.5 ± 0.80 (1.24-1.82)  SRP + M: 1.1 ± 0.68 (.85-1.34)  Mean PPD change:  SRP + P: 0.8 ± 1.07 (0.27-1.37)  SRP + M: 2.1 ± 0.61 (1.80-2.45)  Mean CAL change:  SRP + P: 0.1 ± 0.88 (-0.28-0.63)  SRP + M: 2.7 ± 0.93 (2.25-3.25)  9 months  PI:  SRP + P: 0.7 ± 0.15 (0.65-0.76)  SRP + M: 0.6 ± 0.15 (0.57-0.68)  mSBI:  SRP + P: 1.3 ± 0.86 (1.03-1.66)  SRP + M: 0.7 ± 0.56 (0.55-0.95)  Mean PPD change:  SRP + P: 1.1 ± 1.01 (0.65-1.70)  SRP + M: 3.6 ± 0.79 (3.26-4.11)  Mean CAL change:  SRP + P: 0.8 ± 0.78 (-0.18-0.77)  SRP + M: 4.1 ± 0.02 (3.58-4.67)  IBD: 6 months  SRP + P: 4.6 ± 0.50 (4.52-4.88)  SRP + M: 3.7 ± 0.72 (3.51-4.03)  IBD : 9 months  SRP + P: 4.7 ± 0.50 (4.53-4.90)  SRP + M: 3.5 ± 0.46 (3.34-3.67) | PPD, CAL, IBD, mSBI ( and PI except at 3 months) significantly improved in metformin group (*p*<0.001) compared to placebo group. |
| Pankaj et al., 2018 (70)  12 months | SRP + placebo gel  SRP + rosuvastatin (1.2%) gel  SRP + metformin (1%) gel | PI:  SRP + P: 1.98 ± 0.33  SRP + R: 1.94 ± 0.31  SRP + M: 1.96 ± 0.35  mSBI:  SRP + P: 1.96 ± 0.61  SRP + R: 2.03 ± 0.61  SRP + M: 2.10 ± 0.66  PPD:  SRP + P: 6.80 ± 1.21  SRP + R: 7.30 ± 1.17  SRP + M: 7.40 ± 1.10  CAL:  SRP + P: 6.06 ± 1.17  SRP + R: 6.23 ± 1.01  SRP + M: 6.20 ± 1.12  IBD :  SRP + P: 5.15 ± 0.35  SRP + R: 5.26 ± 0.87  SRP + M: 5.18 ± 0.65 | 6 months  PI:  SRP + P: 0.79 ± 0.13  SRP + R: 0.81 ± 0.11  SRP + M: 0.76 ± 0.12  mSBI:  SRP + P: 1.16 ± 0.59  SRP + R: 0.86 ± 0.73  SRP + M: 0.70 ± 0.65  Mean PPD reduction:  SRP + P: 1.03 ± 0.76  SRP + R: 2.66 ± 0.844  SRP + M: 3.10 ± 1.15  Mean CAL gain:  SRP + P: 1.23 ± 0.90  SRP + R: 2.77 ± 1.14  SRP + M: 3.67 ± 1.03  IBD :  SRP + P: 5.20 ± 0.01  SRP + R: 3.74 ± 0.42  SRP + M: 3.63 ± 0.29  12 months  PI:  SRP + P: 0.59 ± 0.09  SRP + R: 0.53 ± 0.11  SRP + M: 0.53 ± 0.09  mSBI:  SRP + P: 0.83 ± 0.64  SRP + R: 0.50 ± 0.50  SRP + M: 0.46 ± 0.57  Mean PPD reduction:  SRP + P: 1.53 ± 1.07  SRP + R: 3.73 ± 1.28  SRP + M: 4.66 ± 1.21  Mean CAL gain:  SRP + P: 1.47 ± 1.07  SRP + R: 3.90 ± 1.15  SRP + M: 1.17 ± 1.17  IBD :  SRP + P: 5.04 ± 0.38  SRP + R: 3.08 ± 0.31  SRP + M: 3.17 ± 0.24  No 3 months data | PPD, CAL and IBD significantly improved for test groups compared to control group at 6 and 12 months (*p*<0.001), but no differences between test groups. |
| Kurian et al., 2018 (75)  12 months | SRP + placebo gel  SRP + metformin (1%) gel  SRP + aloe vera gel | PI:  SRP + P: 1.98 ± 0.33  SRP + M: 2.13 ± 0.23  SRP + A: 2.13 ± 0.27  BI:  SRP + P: 2.16 ± 0.64  SRP + M: 2.36 ± 0.66  SRP + A: 2.33 ± 0.60  PPD:  SRP + P: 7.63 ± 1.18  SRP + M: 7.96 ± 1.09  SRP + A: 7.51 ± 1.16  CAL:  SRP + P: 6.03 ± 1.18  SRP + M: 6.13 ± 0.86  SRP + A: 6.26 ± 1.11  IBD:  SRP + P: 4.87 ± 0.72  SRP + M: 5.09 ± 0.61  SRP + A: 4.79 ± 0.51 | 6 months  PI:  SRP + P: 1.35 ± 0.28  SRP + M: 1.24 ± 0.17  SRP + A: 1.26 ± 0.27  BI:  SRP + P: 1.56 ± 0.56  SRP + M: 1.43 ± 0.56  SRP + A: 1.46 ± 0.57  Mean PPD reduction:  SRP + P: 0.93 ± 0.69  SRP + M: 3.36 ± 1.27  SRP + A: 1.63 ± 0.80  Mean CAL gain:  SRP + P: 1.06 ± 0.91  SRP + M: 2.57 ± 1.38  SRP + A: 2.16 ± 1.15  IBD:  SRP + P: 4.46 ± 0.72  SRP + M: 3.57 ± 0.48  SRP + A: 4.54 ± 0.53  12 months :  PI:  SRP + P: 1.02 ± 0.22  SRP + M: 0.83 ± 0.17  SRP + A: 0.92 ± 0.20  BI:  SRP + P: 1.36 ± 0.62  SRP + M: 0.83 ± 0.64  SRP + A: 1.06 ± 0.63  Mean PPD reduction:  SRP + P: 1.30 ± 0.87  SRP + M: 3.86 ± 1.27  SRP + A: 2.73 ± 0.98  Mean CAL gain:  SRP + P: 1.43 ± 1.04  SRP + M: 3.73 ± 1.14  SRP + A: 2.63 ± 0.94  IBD:  SRP + P: 4.43 ± 0.73  SRP + M: 3.44 ± 0.40  SRP + A: 4.46 ± 0.50  No 3 months data | Metformin gel and aloe vera gel improved significantly PPD, CAL and IBD compared to placebo gel at 6 and 12 months. Results significantly better for metformin gel compared to aloe vera gel. |

**Supplemental table 18 - Characteristics of the included studies evaluating the effect of gels loaded with bisphosphonates**

| **Study** | **Design; duration** | **Sample size ; mean age; gender (M/F)** | **Clinical parameters evaluated** | **Inclusion criteria** | **Treatment** | **Local administration** | **Findings** |
| --- | --- | --- | --- | --- | --- | --- | --- |
| Sharma & Pradeep, 2012 (79) | RCT  6 months | 66; 30-50; 39/34 | mSBI  PI  PPD  CAL | Chronic periodontitis.  PPD ≥ 5 mm or CAL ≥ 4 to 6 mm and vertical bone loss ≥ 3 mm | SRP + placebo gel  SRP + alendronate (1%) gel | Gel application right after the SRP | Clinical parameters greater for test group compared to control group. |
| Sharma et al., 2017 (80) | RCT  6 months | 46; 30-50; 46/0 | PI  mSBI  PPD  PAL | Chronic periodontitis.  PPD ≥ 5 mm or PAL ≥ 4 to 6 mm and vertical bone loss ≥ 3 mm | SRP + placebo gel  SRP + alendronate (1%) gel | One gel application after the SRP | Mean PPD reduction and PAL gain better in alendronate gel group than placebo group. |
| Dutra et al., 2017 (76) | RCT  6 months | 20; 44.9; 8/12 | PI  BOP  PPD  CAL | Moderate to advanced chronic periodontitis. 2 contralateral teeth, featuring contact with the adjacent teeth, with proximal site showing PPD ≥ 5 and CAL ≥ 3mm associated with proximal vertical bone defects | SRP + placebo gel  SRP + sodium alendronate (1%) gel | One gel application after the SRP | Both treatment produced similar results on PPD, BOP and bone height, but better CAL gain in alendronate group compared to placebo. |
| Pradeep, Kanoriya, et al., 2017 (60) | RCT  9 months | 104; 30-50; 53/51 | mSBI  PI  PPD  CAL | Chronic generalized periodontitis.  PPD ≥ 5 mm or CAL ≥ 4 to 6 mm and vertical bone loss ≥ 3 mm | SRP + placebo  SRP + alendronate (1%) gel  SRP + atorvastatin (1.2%) gel | One application after SRP | PPD, CAL and defect depth reduction (DDR) significantly improved in test groups than in control groups at 3 months. |
| Gupta et al., 2018 (77) | RCT  6 months | 40; 30-50; 23/17 | PI  GI  PPD  CAL | Chronic periodontitis patient. At least one intrabony defect with pocket PPD ≥5 mm or clinical attachment loss (CALs) ≥4 and vertical bone loss ≥3 mm | SRP + placebo  SRP + zoledronate (0.05%) gel | One application after SRP | PPD and CAL significantly improved at 6 months compared to control group. |
| Sheokand et al., 2019 (78) | RCT  6 months | 17; 30-50; NA | PI  GI  PPD CAL | Chronic periodontitis. pocket depth ≥ 5 mm, clinical attachment loss ≥ 3 mm and radiographic evidence of vertical osseous defects 3–6 mm | SRP + placebo gel  SRP + alendronate (1%) gel | One gel application after the SRP | Clinical and radiographical parameters improved in alendronate group compared to placebo group. Alendronate gel more effective in non-smoker than in smoker group. |

**Supplemental table 19 - Periodontal parameters evaluation at baseline and after treatment in bisphosphonates studies**

| **Study** | **Treatments** | **Clinical parameters at baseline** | **Clinical parameters at the end of the treatment** | **Differences between treatment modality** |
| --- | --- | --- | --- | --- |
| Sharma & Pradeep, 2012 (79)  6 months | SRP + placebo gel  SRP + alendronate (1%) gel | PPD:  SRP + P: 7.24 ± 2.18  SRP + A: 7.58 ± 2.13  CAL:  SRP + P: 5.64 ± 1.72  SRP + A: 6.06 ± 1.82  IBD:  SRP + P: 4.71 ± 1.04  SRP + A: 4.70 ± 1.00 | 6 months:  Mean PPD reduction:  SRP + P: 2.15 ± 1.12  SRP + A: 4.48 ± 1.27  Mean CAL gain:  SRP + P: 1.61 ± 0.86  SRP + A: 4.03 ± 0.84  IBD:  SRP + P: 4.60 ± 1.06  SRP + A: 2.82 ± 0.87  No 3 months results | PPD, CAL gain and iBD fill were significantly higher in test group compared to control (*p*<0.001). |
| Sharma et al., 2017 (80)  6 months | SRP + placebo gel  SRP + alendronate (1%) gel | PPD:  SRP + P: 7.62 ± 1.97  SRP + A: 7.84 ± 2.04  PAL:  SRP + P: 6.19 ± 1.54  SRP + A: 6.43 ± 1.77  IBD:  SRP + P: 5.10 ± 0.95  SRP + A: 5.18 ± 1.00 | 6 months:  Mean PPD reduction:  SRP + P: 2.05 ± 0.94  SRP + A: 4.16 ± 1.23  Mean PAL gain:  SRP + P: 1.78 ± 1.22  SRP + A: 3.95 ± 0.88  IBD:  SRP + P: 4.97 ± 0.95  SRP + A: 3.07 ± 0.94  No 3 months results | PPD, PAL, IBD significantly improved (*p*<0.001) at 6 months for SRP + alendronate (1%) gel compared to SRP + placebo gel. |
| Dutra et al., 2017 (76)  6 months | SRP + placebo gel  SRP + sodium alendronate (1%) gel | PPD:  SRP + P: 6.2 ± 1.6  SRP + A: 6.4 ± 1.4  CAL:  SRP + P: 6.7 ± 1.1  SRP + A: 6.9 ± 1.2 | 3 months:  PPD:  SRP + P: 4.4 ± 1.8  SRP + A: 4.3 ± 1.6  CAL:  SRP + P: 5.1 ± 0.9  SRP + A: 4.3 ± 1.1  6 months:  PPD:  SRP + P: 4.4 ± 1.7  SRP + A: 4.3 ± 1.6  CAL:  SRP + P: 4.3 ± 0.7  SRP + A: 3.6 ± 0.7 | No significant differences at 3 and 6 months. |
| Pradeep, Kanoriya, et al., 2017 (60)  9 months | SRP + placebo  SRP + alendronate (1%) gel  SRP + atorvastatin (1.2%) gel | PI:  SRP + P: 1.98 ± 0.334  SRP + AL: 1.95 ± 0.356  SRP + AT: 1.94 ± 0.318  mSBI:  SRP + P: 1.93 ± 0.63  SRP + AL: 2.10 ± 0.66  SRP + AT: 2.00 ± 0.64  PPD:  SRP + P: 6.76 ± 1.22  SRP + AL: 6.96 ± 1.12  SRP + AT: 6.56 ± 1.38  CAL:  SRP + P: 6.13 ± 1.13  SRP + AL: 5.93 ± 0.94  SRP + AT: 6.13 ± 0.89  IBD:  SRP + P: 5.17 ± 0.35  SRP + AL: 5.18 ± 0.65  SRP + AT: 5.46 ± 0.81 | 3 months  PI:  SRP + P: 1.26 ± 0.2  SRP + AL: 1.28 ± 0.225  SRP + AT: 1.29 ± 0.206  mSBI:  SRP + P: 1.43 ± 0.56  SRP + AL: 1.16 ± 0.64  SRP + AT: 1.16 ± 0.59  Mean PPD reduction:  SRP + P: 0.33 ± 0.47  SRP + AL: 1.53 ± 0.50  SRP + AT: 1.06 ± 0.63  Mean CAL gain:  SRP + P: 0.16 ± 0.37  SRP + AL: 1.06 ± 0.52  SRP + AT: 1.66 ± 0.60  9 months  PI:  SRP + P: 0.59 ± 0.092  SRP + AL: 0.54 ± 0.11  SRP + AT: 0.57 ± 0.11  mSBI:  SRP + P: 0.80 ± 0.66  SRP + AL: 0.43 ± 0.50  SRP + AT: 0.40 ± 0.49  Mean PPD reduction:  SRP + P: 1.50 ± 1.07  SRP + AL: 4.30 ± 1.39  SRP + AT: 3.53 ± 1.27  Mean CAL gain:  SRP + P: 1.50 ± 1.07  SRP + AL: 3.50 ± 1.16  SRP + AT: 4.26 ± 1.08  IBD:  SRP + P: 5.04 ± 0.38  SRP + AL: 2.79 ± 0.30  SRP + AT: 3.47 ± 0.70 | CAL and PPD significantly improved in test groups at 3, 6 and 9 months for test groups compared to control group (*p*<0.05).  Alendronate was found to be significantly better than atorvastatin in term of PPD and CAL improvement. |
| Gupta et al., 2018 (77)  6 months | SRP + placebo gel  SRP + zoledronate (0.05%) gel | PI:  SRP + P: 2.24 ± 0.10  SRP + Z: 2.30 ± 0.25  GI:  SRP + P: 2.29 ± 0.13  SRP + Z: 2.32 ± 0.24  PPD:  SRP + P: 7.21 ± 2.64  SRP + Z: 5.90 ± 1.12  CAL:  SRP + P: 7.71 ± 2.55  SRP + Z: 6.25 ± 1.25 | 3 months:  PI:  SRP + P: 1.86 ± 0.06  SRP + Z: 1.94 ± 0.20  GI:  SRP + P: 1.89 ± 0.10  SRP + Z: 1.90 ± 0.14  PPD:  SRP + P: 7.00 ± 2.35  SRP + Z: 5.55 ± 0.89  CAL:  SRP + P: 7.50 ± 2.44  SRP + Z: 5.90 ± 1.07  6 months :  PI:  SRP + P: 1.63 ± 0.11  SRP + Z: 1.64 ± 0.17  GI:  SRP + P: 1.63 ± 0.11  SRP + Z: 1.62 ± 0.16  PPD:  SRP + P: 6.86 ± 2.28  SRP + Z: 4.75 ± 0.79  CAL:  SRP + P: 7.43 ± 2.38  SRP + Z: 5.10 ± 0.97 | PPD and CAL significantly improved in test group compared to control group at 6 months only (p<0.05). |
| Sheokand et al., 2019 (78)  6 months | SRP + placebo gel  SRP + alendronate (1%) gel | PI: smoker  SRP + P: 1.55 ± 0.32  SRP + A: 1.55 ± 0.39  GI: smoker  SRP + P: 1.50 ± 0.39  SRP + A: 1.55 ± 0.32  PPD: smoker  SRP + P: 7.00 ± 0.93  SRP + A: 7.07 ± 0.96  CAL: smoker  SRP + P: 7.60 ± 1.24  SRP + A: 7.80 ± 1.15  PI: non-smoker  SRP + P: 1.93 ± 0.54  SRP + A: 1.93 ± 0.49  GI: non-smoker  SRP + P: 1.88 ± 0.51  SRP + A: 1.93 ± 0.49  PPD: non-smoker  SRP + P: 6.93 ± 0.80  SRP + A: 7.33 ± 0.98  CAL: non-smoker  SRP + P: 7.80 ± 1.01  SRP + A: 8.13 ± 1.13 | 3 months  PI: smoker  SRP + P: 1.17 ± 0.35  SRP + A: 1.18 ± 0.42  GI: smoker  SRP + P: 1.10 ± 0.42  SRP + A: 1.17 ± 0.35  PPD: smoker  SRP + P: 5.47 ± 0.99  SRP + A: 5.87 ± 0.74  CAL: smoker  SRP + P: 6.00 ± 1.13  SRP + A: 6.60 ± 0.91  PI: non-smoker  SRP + P: 1.33 ± 0.44  SRP + A: 1.35 ± 0.44  GI: non-smoker  SRP + P: 1.22 ± 0.57  SRP + A: 1.35 ± 0.44  PPD: non-smoker  SRP + P: 4.53 ± 0.83  SRP + A: 5.47 ± 0.99  CAL: non-smoker  SRP + P: 5.40 ± 0.99  SRP + A: 6.27 ± 1.10  6 months:  PI: smoker  SRP + P: 0.55 ± 0.14  SRP + A: 0.60 ± 0.16  GI: smoker  SRP + P: 0.58 ± 0.15  SRP + A: 0.55 ± 0.14  PPD: smoker  SRP + P: 4.73 ± 0.88  SRP + A: 4.60 ± 0.83  CAL: smoker  SRP + P: 5.73 ± 0.88  SRP + A: 5.33 ± 1.05  PI: non-smoker  SRP + P: 0.62 ± 0.40  SRP + A: 0.50 ± 0.34  GI: non-smoker  SRP + P: 0.63 ± 0.41  SRP + A: 0.57 ± 0.33  PPD: non-smoker  SRP + P: 3.27 ± 0.27  SRP + A: 3.07 ± 0.70  CAL: non-smoker  SRP + P: 4.13 ± 0.83  SRP + A: 3.87 ± 0.83 | PPD and CAL significantly improved in test group compared to control group, in non-smoker patients (*p*<0.05) only at 3 months. |

**Supplemental table 20 - Characteristics of the included studies evaluating the effect of gels loaded with other drugs**

| **Study** | **Design; duration** | **Sample size ; mean age; gender (M/F)** | **Clinical parameters evaluated** | **Inclusion criteria** | **Treatment** | **Local administration** | **Findings** |
| --- | --- | --- | --- | --- | --- | --- | --- |
| R. V. Chandra et al., 2013 (89) | RCT  6 months | 31; 33.9 ± 7.66; 20/11 | PI  mGI  PPD  CAL | At least 4 periodontal pockets ≥ 5 mm with at least 1 pocket in each quadrant showing radiographic evidence of bone loss ≥ 3 mm | SRP  SRP + placebo  SRP + lycopene gel (2%) | Gel application after the SRP | Lycopene gel application was effective to reduce gingival inflammation and to increase CAL. |
| Elgendy et al., 2013 (86) | RCT  6 months | 40; 30-60; 21/19 | PI  GI  PPD  CAL | Untreated moderate to severe chronic or recurrent periodontitis  Single rooted teeth with probing pocket depth (PPD) of 5-8 mm, without any recession + BOP | SRP  SRP + tee tree oil (TTO) (5%) gel | Gel application after SRP | TTO gel application improved periodontal outcomes vs SRP alone. |
| Warad et al., 2013 (88) | RCT  3 months | 15; 18-55 (43.9 ± 5.4); NA | GI  PPD  RAL | Moderate to severe chronic periodontitis.  At least four isolated PPD with a probing depth between 5 and 8 mm | SRP  SRP + lemongrass (2%) essential oil gel | Gel application after the SRP | Clinical parameters (PPD, GI and RAL) significantly improved at 1 and 3 months. |
| Phogat et al., 2014 (30) | RCT  3 months | 30; 30-50; NA | PI  GI  PPD  CAL | Chronic periodontitis with at least 3 nonadjacent interproximal sites with 4-8 mm of PPD | SRP  SRP + chlosite gel  SRP + herbal gel  Chlosite gel  Herbal gel | Gels application 3 times: after the SRP, and 2 times more with an interval of 10 days | Significant improvement of clinical parameters for SRP + chlosite gel and SRP + herbal gel compared with SRP. |
| Rajan et al., 2014 (81) | RCT  3 months | 33; NA ; 15/18 | GI  PI  BOP  PPD  CAL | Moderate to severe chronic periodontitis with ≥ 5 sites of PPD ≥ 5 mm having a minimum of 20 teeth remaining | SRP  SRP + hyaluronan (0.2%) gel | Gel application after the SRP and 7 days after | Adjunctive injection of HA improved periodontal parameters at 3 months. |
| Yaghini et al., 2014 (91) | RCT  3 months | 18; 31-52 ; NA | PPD  CAL  PBI  PI | Moderate chronic periodontitis. ≥ 4 periodontal pockets with an average depth of 3-5mm | SRP + placebo gel  SRP + herbal gel | SRP, then 2 weeks later 2^nd^ SRP if needed and gel application. One week after: second gel application | Significant improvement of clinical parameters for both groups, but no differences between them. |
| Polepalle et al., 2015 (82) | RCT  3 months | 18; 30-60; 11/7 | PI  BOP  PPD  CAL | Included ≥ 20 teeth present with at least 5 inter proximal  sites with probing depth (PPD) ≥ 5 mm | SRP  SRP + hyaluronan (0.8%) gel | Gel application after the SRP and 7 days later | Adjunctive injection of hyaluronan (0.8%) gel improved periodontal and microbiological parameters. |
| Debnath et al., 2016 (92) | RCT  3 months | 6; 30-60; NA | PI  GI  PBI  PPD  CAL | A minimum of 20 teeth present and a probing depth of 5–7 mm classified as localized/generalized chronic periodontitis | SRP  SRP + NBF gel | One application after SRP | Significant improvement of both clinical and microbiological parameters. |
| Grover et al., 2016 (87) | RCT  3 months | 46; 30-58 ; 22/18 | PI  GI  mSBI  PPD  CAL | Chronic periodontitis. ≥ 30 years old, having ≥ 20 teeth and at least 8 sites with PPD ≥ 5 mm | SRP + placebo gel  SRP + (10%) *E. officinalis* gel | One application after SRP | Adjunctive use of 10%  *E. officinalis* gel leads to significantly greater improvement than placebo group. |
| Shah et al., 2016 (83) | RCT  3 months | 9; 30-60; NA | PI  GI  PPD  RAL | Chronic periodontitis, with probing pocket  depth (PPD) ≥ 5 mm in at least 1 tooth in the contralateral quadrants | SRP  SRP + hyaluronan (0.8%) gel | Gel application after SRP and 1 week after | Clinical parameters significantly improved in test group compared to control group. |
| Rattanasuwan et al., 2016 (84) | RCT  6 months | 48; 34-74; 22/20 | PPD  CAL  GI  BOP  FMPS | Chronic periodontitis. PPD of 5–10 mm without furcation involvement | SRP + placebo gel  SRP + green tea gel | Gel application after the SRP, at 7 and 14d | BOP and GI significantly reduced in test group compared to control group at 3 months. No change for PPD and CAL. |
| Mahendra et al., 2017 (93) | RCT  3 months | 50; 30-65; 27/23 | PPD  CAL  PI  BI | Generalized  chronic periodontitis with PPD ≥ 5 mm | SRP+ *Garcinia mangostana*  (4%) gel  SRP + placebo | One gel application after the SRP | Significant improvement for SRP + Garcinia mangostana vs SRP + placebo. |
| Kanoriya et al., 2018 (94) | RCT  6 months | 42; 25-55; NA | Defect depth reduction (DDR)  PI  mSBI  PPD  CAL | Chronic periodontitis. PPD ≥ 5 mm or CAL ≥ 4 mm and vertical bone loss ≥ 3 mm | SRP + placebo gel  SRP + boric acid gel (0.75 mg/0.1 mL) | One gel application after the SRP | Significant improvement of PPD and CAL for test group compared to control group. |
| Rayyan et al., 2018 (99) | RCT  6 months | 5; 43.5 ± 7.9 ; NA | PI  GI  PPD  BOP | Chronic periodontitis. PPD ≥5 mm with evident radiographic bone loss | SRP + placebo gel  SRP + grape seed extract gel | Gels application at day 0, 3, 6 and 9 after the baseline. SRP realized 1 week before the baseline | GI and PI significantly improved at 6 months. |
| Kaur et al., 2019 (95) | RCT  3 months | 29;20-65; 20/9 | PI  BI  PPD  RAL | Moderate to severe chronic generalized periodontitis with ≥4 sites with PPD ≥5 mm in one or both of arches | SRP  SRP + curcumin (1%) gel | One gel application after the SRP | No statistical differences between test and control group at 3 months. |
| Pranam et al., 2020 (96) | RCT  3 months | 16; 30-50; NA | PI  GI  PPD | Moderate chronic periodontitis, with PPD of 5 to 7 mm in four nonadjacent interproximal sites | SRP + placebo gel  SRP + Coenzyme Q10 (CoQ10) gel | One gel application after the SRP | No statistical differences between test and control group at 3 months. |
| Taalab et al., 2021 (85) | RCT  6 months | 30; 25-50; 10/20 | BOP  GI  PPD  CAL | Stage II generalized periodontitis | SRP  SRP + tea tree oil (TTO) (5%) gel | Gel application after the SRP | Except GI, no clinical parameters improved at 3 months. |
| Aslroosta et al., 2021 (97) | RCT  3 months | 30; 32-68; 14/16 | PI  GI  BI  PPD  CAL | Stage III periodontitis. CAL of ≥5 mm and PPD ≥6 mm with bleeding on probing around ≥2 non‐adjacent teeth | SRP  SRP + erythropoietin  (EPO)(4000 units) gel | Gel application after the SRP and repeated 5 times every other day | All clinical parameters significantly improved. PPD and CAL significantly improved compared to control group. |
| Ahmed et al., 2021 (98) | RCT  3 months | 24; 32-55; 7/17 | PI  GI  PPD  CAL | Stage II periodontitis | SRP + placebo gel  SRP + melatonin gel | Gel application after the SRP and weekly once for four weeks | PPD and CAL significantly improved compared to control group. |

**Supplemental table 21 - Periodontal parameters evaluation at baseline and after treatment in other drugs studies**

| **Study** | **Treatments** | **Clinical parameters at baseline** | **Clinical parameters at the end of the treatment** | **Differences between treatment modality** |
| --- | --- | --- | --- | --- |
| R. V. Chandra et al., 2013 (89)  6 months | SRP  SRP + placebo  SRP + lycopene (L) gel (2%) | PPD:  SRP: 5.88 + 1.28  SRP + placebo: 5.66 + 1.17  SRP + L: 6.07 + 1.07  CAL:  SRP: 6.22 + 1.28  SRP + placebo: 5.58 + 1.32  SRP + L: 6.55 + 1.15  PI:  SRP: 3.67 + 0.59  SRP + placebo: 3.68 + 0.6  SRP + L: 3.55 + 0.57  MGI:  SRP: 2.94 + 0.36  SRP + placebo: 2.93 + 0.36  SRP + L: 2.92 + 0.43 | 3 months  PPD:  SRP: 5.03 + 1.28  SRP + placebo: 4.55 + 1.18  SRP + L: 4.40 + 1.04  CAL:  SRP: 5.48 + 1.22  SRP + placebo: 4.81 + 1.14  SRP + L: 4.62 + 1.07  PI:  SRP: 2.23 + 0.77  SRP + placebo: 1.58 + 0.49  SRP + L: 1.79 + 0.55  MGI:  SRP: 0.99 + 0.61  SRP + placebo: 0.91 + 0.65  SRP + L: 0.81+0.47  6 months:  PPD:  SRP: 4.92 + 1.35  SRP + placebo: 4.51 + 1.22  SRP + L: 3.62 + 1.04  CAL:  SRP: 5.14 + 1.45  SRP + placebo: 4.77 + 1.25  SRP + L: 3.77 + 1.01  PI:  SRP: 1.77 + 0.46  SRP + placebo: 1.73 + 0.54  SRP + L: 1.68 + 0.60  MGI:  SRP: 1.36 + 0.37  SRP + placebo: 0.90 + 0.62  SRP + L: 0.79 + 0.47 | Significant difference at 6 months for PPD and CAL between SRP + L vs SRP + placebo but no differences between them at 3 months.  Significant difference for PI between SRP+L vs SRP + placebo was observed at 3 months while no differences were found at 3 and 6 months for MGI. |
| Elgendy et al., 2013 (86)  6 months | SRP  SRP + tea tree oil (TTO) gel | PI:  SRP: 1.86 + 0.52  SRP + TTO: 1.76 + 0.51  GI:  SRP: 2.17 + 0.4  SRP + TTO: 2.01 + 0.49  PPD:  SRP: 5.3 + 0.47  SRP + TTO: 5.25 + 0.44  CAL:  SRP: 4.9 + 0.78  SRP + TTO: 5 + 0.79 | 3 months  PI:  SRP: 0.53 + 0.23  SRP + TTO: 0.48 + 0.18  GI:  SRP: 0.65 + 0.17  SRP + TTO: 0.37 + 0.2  PPD:  SRP: 3.8 + 0.41  SRP + TTO: 3.3 + 0.47  CAL:  SRP: 3.6 + 0.59  SRP + TTO: 3.2 + 0.52  6 months:  PI:  SRP: 0.50 + 0.21  SRP + TTO: 0.41 + 0.15  GI:  SRP: 0.66 + 0.26  SRP+TTO: 0.35 + 0.17  PPD:  SRP: 3.90 + 0.44  SRP + TTO: 3.40 + 0.50  CAL:  SRP: 3.70+0.57  SRP + TTO: 3.25+0.41 | GI, PPD, CAL were significantly improved in SRP + TTO group vs SRP alone at 3 and 6 months. |
| Warad et al., 2013 (88)  3 months | SRP  SRP + lemongrass (2%) essential oil gel | PPD:  SRP: 6.33 ± 0.95  SRP + L: 6.60 ± 0.81  RAL:  SRP: 9.43 ± 0.97  SRP + L: 9.83 ± 0.94  GI:  SRP: 1.99 ± 0.76  SRP + L: 1.99 ± 0.61 | PPD:  SRP: 4.93 ± 0.90  SRP + L: 4.20 ± 0.71  RAL:  SRP: 7.46 ± 1.16  SRP + L: 6.63 ± 0.80  GI:  SRP: 1.70 ± 0.40  SRP + L: 1.63 ± 0.75 | Significant improvement for PPD, RAL gain and GI at 3 months. |
| Phogat et al., 2014 (30)  3 months | SRP  SRP + chlosite gel (1.5%)  SRP + herbal gel  Chlosite gel (1.5%)  Herbal gel |  | Mean changes at 3 months  PI:  SRP: 1.134 ± 0.144  SRP + C: 2.245 ± 0.191  SRP + H: 1.785 ± 0.001  GI:  SRP: 1.231 ± 0.001  SRP + C: 2.281 ± 0.212  SRP + H: 1.864 ± 0.105  PPD:  SRP: 2.264 ± 0.031  SRP + C: 3.764 ± 0.010  SRP + H: 2.917 ± 0.082  CAL:  SRP: 2.405 ± 0.079  SRP + C: 2.913 ± 0.051  SRP + H: 2.784 ± 0.056 | Significant differences between SRP + chlosite gel/ SRP + herbal gel compared to SRP alone in term of PI, GI, PPD, and CAL (*p*<0.001). |
| Rajan et al., 2014 (81)  3 months | SRP  SRP + hyaluronan gel | BI:  SRP: 1.00 ± 0.00  SRP + H: 1.00 ± 0.00  PPD:  SRP: 6.09 ± 1.26  SRP + H: 6.33 ± 0.99  CAL:  SRP: 9.12 ± 1.67  SRP + H: 10.18 ± 2.08 | BI:  SRP: 0.48 ± 0.51  SRP + H: 0.06 ± 0.24  Mean PPD reduction:  SRP: 1.73 ± 1.04  SRP + H: 3.85 ± 1.06  Mean CAL gain:  SRP: 1.63 ± 0.55  SRP + H: 3.27 ± 1.64 | Mean differences between control and test group were significant for PPD , CAL and BI (*p*<0.001). |
| Yaghini et al., 2014 (91)  3 months | SRP + placebo gel  SRP + herbal gel | PPD:  SRP + P: 4.15 (0.54)  SRP + H: 4.08 (0.60)  CAL:  SRP + P: 3.1 (0.64)  SRP + H: 3.05 (0.47) | PPD:  SRP + P: 2.57 (1)  SRP + H: 2.44 (0.87)  CAL:  SRP + P: 1.18 (0.92)  SRP + H: 0.8 (0.82) | Statistical differences for both group from baseline to 3 months, but no differences found between groups. |
| Polepalle et al., 2015 (82)  3 months | SRP  SRP + hyaluronan (0.8%) gel (H) | BOP:  SRP: 1.00 ± 0.00  SRP + H: 1.00 ± 0.00  PI:  SRP: 2.15 ± 0.23  SRP + H: 2.05 ± 0.32  PPD:  SRP: 5.21 ± 0.54  SRP + H: 4.99 ± 0.34  CAL:  SRP: 5.41 ± 0.65  SRP + H: 5.4 ± 0.71 | BOP:  SRP: 0.92 ± 0.09  SRP + H: 0.25 ± 0.07  PI:  SRP: 1.90 ± 0.25  SRP + H: 0.40 ± 0.11  PPD:  SRP: 4.49 ± 0.47  SRP + H: 2.45 ± 0.31  CAL:  SRP: 4.71 ± 0.64  SRP + H: 2.68 ± 0.57 | Both periodontal (BOP, PI, PPD and CAL) and microbiological parameters significantly improved (*p*<0.001). |
| Debnath et al., 2016 (92)  3 months | SRP  SRP + Nano-Bio Fusion (NBF) gel | PI:  SRP: 1.14 ± 0.14  SRP + N: 1.17 ± 0.14  GI:  SRP: 1.3 ± 0.27  SRP + N: 1.28 ± 0.23  SBI:  SRP: 1.41 ± 0.48  SRP + N: 1.28 ± 0.27  PPD:  SRP: 5.79 ± 0.78  SRP + N: 5.7 ± 0.72  CAL:  SRP: 4.76 ± 0.79  SRP + N: 4.7 ± 0.72 | PI:  SRP: 0.11 ± 0.14  SRP + N: 0.09 ± 0.12  GI:  SRP: 0.07 ± 0.11  SRP + N: 0.07 ± 0.12  SBI:  SRP: 0.09 ± 0.14  SRP + N: 0.06 ± 0.11  PPD:  SRP: 2.95 ± 0.32  SRP + N: 2.15 ± 0.36  CAL:  SRP: 1.95 ± 0.32  SRP + N: 1.15 ± 0.36 | Significant improvement of PPD (*p*=0.001) and CAL (*p*=0.01) for test group compared to control group. |
| Grover et al., 2016 (87)  3 months | SRP + placebo gel  SRP + + (10%) *E. officinalis* gel | PI:  SRP + P: 1.885 ± 0.226  SRP + O: 1.955 ± 0.280  GI:  SRP + P: 2.636 ± 0.273  SRP + O: 2.599 ± 0.302  PPD:  SRP + P: 3.472 ± 0.497  SRP + O: 3.623 ± 0.688  CAL:  SRP + P: 4.722 ± 1.157  SRP + O: 4.347 ± 1.159  mSBI:  SRP + P: 2.682 ± 0.344  SRP + O: 2.558 ± 0.356 | Mean changes  PI:  SRP + P: 1.163 ± 0.404  SRP + O: 1.149 ± 0.380  GI:  SRP + P: 1.194 ± 0.296  SRP + O: 1.263 ± 0.328  Mean PPD reduction:  SRP + P: 0.81 ± 0.33  SRP + O: 1.42 ± 0.60  Mean CAL gain:  SRP + P: 1.03 ± 0.49  SRP + O: 1.69 ± 0.81  mSBI:  SRP + P: 1.903 ± 0.471  SRP + O: 2.205 ± 0.270 | Significant improvement were observed in test group compared to control groups at 3 months for PPD , CAL and mSBI (*p*<0.05). |
| Shah et al., 2016 (83)  3 months | SRP  SRP + hyaluronan (0.8%) gel | PI:  SRP: 2.33 ± 0.25  SRP + N: 2.41 ± 0.19  GI:  SRP: 2.28 ± 0.20  SRP + N: 2.36 ± 0.18  PPD:  SRP: 5.37 ± 0.56  SRP + N: 5.37 ± 0.56  RAL:  SRP: 9.10 ± 2.50  SRP + N: 9.17 ± 2.04 | PI:  SRP: 1.82 ± 0.31  SRP + N: 1.81 ± 0.44  GI:  SRP: 1.76 ± 0.23  SRP + N: 1.74 ± 0.32  PPD:  SRP: 3.33 ± 0.92  SRP + N: 2.37 ± 0.61  RAL:  SRP: 7.50 ± 2.58  SRP + N: 6.70 ± 1.64 | Significant PPD and RAL improvement (*p*<0.05) for test group compared to control group. |
| Rattanasuwan et al., 2016 (84)  6 months | SRP + placebo gel  SRP + green tea | PPD:  SRP + P: 5.50 ± 0.91  SRP + T: 5.84 ± 1.11  CAL:  SRP + P: 5.78 ± 1.36  SRP + T: 6.08 ± 1.68  GI:  SRP + P: 2.00 ± 0.33  SRP + T: 1.95 ± 0.22 | 3 months :  PPD:  SRP + P: 3.02 ± 1.05  SRP + T: 3.19 ± 1.11  CAL:  SRP + P: 3.74 ± 1.63  SRP + T: 3.87 ± 1.60  GI:  SRP + P: 0.79 ± 0.86  SRP + T: 0.55 ± 0.76  6 months :  PPD:  SRP + P: 2.83 ± 1.14  SRP + T: 3.13 ± 1.20  CAL:  SRP + P: 3.61 ± 1.70  SRP + T: 3.86 ± 1.70    GI:  SRP + P:0.75 ± 0.82  SRP + T: 0.78 ± 0.79 | No statistical differences for PPD and CAL between group. GI significantly improved in test group compared to control group at 1 and 3 months. |
| Mahendra et al., 2017 (93)  3 months | SRP + *Garcinia mangostana (G)*  (4%) gel  SRP + placebo | PPD:  SRP + placebo: 6.27 + 1.20  SRP + G: 6.65 + 1.207  CAL:  SRP + placebo: 6.49 + 1.32  SRP + G: 6.99 + 1.3  PI:  SRP + placebo: 2.001 + 0.41  SRP + G: 1.991 + 0.34  BI:  SRP + placebo: 92.15 + 10  SRP + G: 91.3 + 10 | PPD:  SRP + placebo: 4.11 + 0.6  SRP + G: 2.9 + 0.52  CAL:  SRP + placebo: 4.32 + 1.03  SRP + G: 3.1 + 0.88  PI:  SRP + placebo: 1.08 + 0.22  SRP + G: 1.04 + 0.32  BI:  SRP + placebo: 53.76 + 10.63  SRP + G: 34.27 + 7.99 | PPD, CAL, BI were significantly improved in SRP + G group vs placebo (*p*<0.001). |
| Kanoriya et al., 2018 (94)  6 months | SRP + placebo gel  SRP + boric acid gel (0.75 mg/0.1 mL) | PI:  SRP + P: 1.84 ± 0.42  SRP + B: 1.98 ± 0.32  mSBI:  SRP + P: 2.10 ± 0.65  SRP + B: 2.15 ± 0.65  PPD:  SRP + P: 6.94 ± 0.91  SRP + B: 6.75 ± 0.85  CAL:  SRP + P: 5.78 ± 1.08  SRP + B: 5.70 ± 0.65 | 3 months  PI:  SRP + P: 1.31 ± 0.30  SRP + B: 1.37 ± 0.18  mSBI:  SRP + P: 1.52 ± 0.61  SRP + B: 1.31 ± 0.67  Mean PPD reduction:  SRP + P: 0.89 ± 0.31  SRP + B: 1.85 ± 0.48  Mean CAL gain:  SRP + P: 0.68 ± 0.47  SRP + B: 1.45 ± 0.51  6 months:  PI:  SRP + P: 0.71 ± 0.11  SRP + B: 0.67 ± 0.14  mSBI:  SRP + P: 1.21 ± 0.53  SRP + B: 0.65 ± 0.49  Mean PPD reduction:  SRP + P: 1.89 ± 0.45  SRP + B: 3.15 ± 0.74  Mean CAL gain:  SRP + P: 1.31 ± 0.82  SRP + B: 2.65 ± 0.58  IBD:  SRP + P: 0.14 ± 0.05  SRP + B: 1.83 ± 0.27 | Significant PPD reduction and CAL gain in SRP + B group compared to SRP + placebo group at 3 and 6 months. Significant improvement of mSBI values at 6 months for boric acid group. |
| Rayyan et al., 2018 (99)  6 months | SRP + placebo gel  SRP + grape seed extract gel | PI:  SRP + P: 2.00 ± 0.39  SRP + G: 1.53 ± 0.64  GI:  SRP + P: 1.55 ± 0.50  SRP + G: 1.69 ± 0.55  PPD:  SRP + P: 5.58 ± 0.71  SRP + G: 5.69 ± 1.31 | 6 months:  PI:  SRP + P: 0.80 ± 0.76  SRP + G: 0.57 ± 0.65  GI:  SRP + P: 1.15 ± 0.83  SRP + G: 0.90 ± 0.74  PPD:  SRP + P: 4.93 ± 1.25  SRP + G: 5.04 ± 1.72  No 3 months data | Significant difference (*p*<0.05) only for PI and GI at 6 months. |
| Kaur et al., 2019 (95)  3 months | SRP  SRP + curcumin (1%) gel | PI:  SRP: 1.96 ± 0.54  SRP + C: 1.59 ± 0.59  BI:  SRP: 1.13 ± 0.57  SRP + C: 1.44 ± 0.76  PPD:  SRP: 3.77 ± 0.88  SRP + C: 3.90 ± 0.49  RAL:  SRP: 8.37 ± 1.13  SRP + C: 8.92 ± 1.38 | PI:  SRP: 1.06 ± 0.44  SRP + C: 0.89 ± 0.36  BI:  SRP: 0.49 ± 0.43  SRP + C: 0.53 ± 0.37  PPD:  SRP: 2.83 ± 0.61  SRP + C: 2.90 ± 0.52  RAL:  SRP: 7.03 ± 1.17  SRP + C: 6.90 ± 1.19 | No statistical difference between the two groups at 3 months. |
| Pranam et al., 2020 (96)  3 months | SRP + placebo  SRP + Coenzyme Q10 (CoQ10) gel | PI:  SRP + P: 2.28 ± 0.74  SRP + CoQ10: 2.30 ± 0.61  GI:  SRP + P: 1.81 ± 0.51  SRP + CoQ10: 1.79 ± 0.46  PPD:  SRP + P: 5.13 ± 0.42  SRP + CoQ10: 5.47 ± 0.56 | PI:  SRP + P: 0.85 ± 0.37  SRP + CoQ10: 0.60 ± 0.16  GI:  SRP + P: 0.85 ± 0.39  SRP + CoQ10: 0.59 ± 0.23  PPD:  SRP + P: 4.03 ± 0.31  SRP + CoQ10: 4.31 ± 0.47 | No statistical difference between the two groups at 3 months. |
| Taalab et al., 2021 (85)  6 months | SRP  SRP + tea tree oil (TTO) (5%) gel | BOP:  SRP: 85 ± 12.7  SRP + TTO: 81.7 ± 22.1  GI:  SRP: 1.07 ± 0.36  SRP + TTO: 1.17 ± 0.48  PPD:  SRP: 5.5 ± 1.1  SRP + TTO: 5.4 ± 1.1  CAL:  SRP: 3.4 ± 0.5  SRP + TTO: 3.3 ± 0.5 | 3 months:  BOP:  SRP: 41.7 ± 32.3  SRP + TTO: 26.7 ± 25.8  GI:  SRP: 0.45 ± 0.40  SRP + TTO: 0.27 ± 0.26  PPD:  SRP: 4.3 ± 1.5  SRP + TTO: 3.3 ± 0.6  CAL:  SRP: 1.7 ± 0.6  SRP + TTO: 1.4 ± 0.5  6 months:  BOP:  SRP: 26.7 ± 20  SRP + TTO: 3.3 ± 8.8  GI:  SRP: 0.27 ± 0.20  SRP + TTO: 0.03 ± 0.09  PPD:  SRP: 2.6 ± 0.6  SRP + TTO: 1.9 ± 0.9  CAL:  SRP: 1.2 ± 0.7  SRP + TTO: 0.4 ± 0.5 | No statistical differences between groups at 3 months (*p*<0.05). Significant improvement of CAL and GI for test group compared control group at 6 months. |
| Aslroosta et al., 2021 (97)  3 months | SRP  SRP + erythropoietin (EPO) gel | PI:  SRP: 2.53 ± 0.89  SRP + EPO: 2.71 ± 0.46  GI:  SRP: 2.6 ± 0.72  SRP + EPO: 2.75 ± 0.75  PPD:  SRP: 3.72 ± 0.72  SRP + EPO: 3.94 ± 0.78  CAL:  SRP: 5.1 ± 4.1  SRP + EPO: 5.67 ± 4.32 | PI:  SRP: 1.1 ± 0.79  SRP + EPO: 1.1 ± 0.68  GI:  SRP: 0.98 ± 0.58  SRP + EPO: 1.33 ± 0.07  PPD:  SRP: 1.95 ± 0.76  SRP + EPO: 2.55 ± 0.86  CAL:  SRP: 3.40 ± 2.71  SRP + EPO: 4.33 ± 3.19 | Significant improvement at 3 months for test group for PPD (p=0.004),CAL (p=0.002) and GI (p=0.002). |
| Ahmed et al., 2021 (98)  3 months | SRP + placebo gel  SRP + melatonin (M) gel | PI:  SRP + placebo: 1.96 ± 0.6  SRP + M: 2 ± 0.7  GI:  SRP + placebo: 2.4 ± 0.5  SRP + M: 2.5 ± 0.5  PPD:  SRP + placebo: 4.0 ± 0.6  SRP + M: 4.3 ± 0.8  CAL:  SRP + placebo: 4.3 ± 0.6  SRP + M: 4.7 ± 0.9 | PI:  SRP + placebo: 0.7 ± 0.6  SRP + M: 0.7 ± 0.6  GI:  SRP + placebo: 0.7 ± 0.5  SRP + M: 0.6 ± 0.5  PPD:  SRP + placebo: 3.1 ± 0.7  SRP + M: 2.9 ± 0.7  CAL:  SRP + placebo: 3.7 ± 0.6  SRP + M: 3.5 ± 0.6 | Significant improvement of PI, GI, PPD and CAL change at 3 months (p<0.001). |
